# Rapid Methods for Antimicrobial Resistance Diagnostics

**DOI:** 10.3390/antibiotics10020209

**Published:** 2021-02-20

**Authors:** Georgia D. Kaprou, Ieva Bergšpica, Elena A. Alexa, Avelino Alvarez-Ordóñez, Miguel Prieto

**Affiliations:** 1 Department of Food Hygiene and Technology, University of León, 24071 León, Spain; ieva.bergspica@bior.lv (I.B.); ealexandra.alexa@gmail.com (E.A.A.); aalvo@unileon.es (A.A.-O.); miguel.prieto@unileon.es (M.P.); 2 Luxembourg Centre for Systems Biomedicine, University of Luxembourg, L-4367 Belvaux, Luxembourg; 3 Institute of Food Safety, Animal Health and Environment BIOR, LV-1076 Riga, Latvia; 4 Institute of Food Science and Technology, University of León, 24071 León, Spain

**Keywords:** molecular diagnostics, antimicrobial resistance, antibiotic susceptibility testing, microfluidics, point-of-care, lab-on-a-chip, MALDI-TOF, FTIR, sequencing

## Abstract

Antimicrobial resistance (AMR) is one of the most challenging threats in public health; thus, there is a growing demand for methods and technologies that enable rapid antimicrobial susceptibility testing (AST). The conventional methods and technologies addressing AMR diagnostics and AST employed in clinical microbiology are tedious, with high turnaround times (TAT), and are usually expensive. As a result, empirical antimicrobial therapies are prescribed leading to AMR spread, which in turn causes higher mortality rates and increased healthcare costs. This review describes the developments in current cutting-edge methods and technologies, organized by key enabling research domains, towards fighting the looming AMR menace by employing recent advances in AMR diagnostic tools. First, we summarize the conventional methods addressing AMR detection, surveillance, and AST. Thereafter, we examine more recent non-conventional methods and the advancements in each field, including whole genome sequencing (WGS), matrix-assisted laser desorption/ionization time-of-flight (MALDI-TOF) spectrometry, Fourier transform infrared (FTIR) spectroscopy, and microfluidics technology. Following, we provide examples of commercially available diagnostic platforms for AST. Finally, perspectives on the implementation of emerging concepts towards developing paradigm-changing technologies and methodologies for AMR diagnostics are discussed.

## 1. Introduction

Antimicrobial Resistance (AMR) has become one of the dominant health challenges of our times. Antibiotic resistance occurs as a natural evolutionary process in bacteria, but can be accelerated by a number of factors [1,2]. More specifically, the excessive and inadequate use of antibiotics in both humans and animals leads to the wide spread of resistant bacteria and their antimicrobial resistant genes (ARGs) [3,4,5]. AMR has severe adverse effects on humans, healthcare systems, farm animals, agriculture, environmental health, and, consequently, on national economies [6]. AMR is a challenging threat undermining key features of current medical care at enormous costs in terms of patient mortality and morbidity, but also in terms of patient treatment expenses [7,8]. Modern, mainstream antibiotic therapeutic strategies are responsible for their own regression by actively selecting for resistant strains, compelling the need for supporting the continuous discovery of new antibiotics in order to remain ahead of the AMR challenge [9]. Therefore, it is urgent to prolong the lifespan of current antibiotics while research and development of new-generation antibiotics takes its course. In addition, it is important to implement efficient control measures for antibiotic use in order to slow down the need for continuous discovery of new antibiotics [1].

The costs related to the soaring AMR rates are forecasted to grow dramatically if no measures are taken [10]. The lack of effective antimicrobials is leading to common infections becoming life-threatening, hence, rendering treatments, such as chemotherapy and surgical procedures being more prone to becoming life-threatening due to common infections. Thus, constraining the misuse and the overuse of antimicrobials is crucial for impeding the dispersion of AMR. According to recent studies [10,11,12,13] more than 33,000 people die every year in the European Union (EU) as a result of infections stemming from antibiotic-resistant bacteria. The annual economic burden related to AMR in the EU is considerable, accounting for an estimated 1.5 billion euros, including healthcare costs and productivity losses [10]. Globally, antibiotic resistance is responsible for more than 500,000 deaths every year, from which more than 40% involve infant deaths [14].

Early detection of pathogens is required for the optimal treatment of infectious diseases. Although great leaps have been made in medical technology, the turnaround time (TAT), both for the detection and the characterization of microbial pathogens, often takes up to several days [15,16,17]. As a result, clinicians are pushed to start empiric antibiotic therapies, typically broad-spectrum, before a diagnosis can be reached. This practice may lead to detrimental consequences not only for the health of the patient (i.e. microbiome dysbiosis), but also for the exacerbation of the ongoing AMR challenge. Thus, the need for rapid, highly sensitive, affordable, and cost-effective detection platforms for AMR diagnostics has become urgent. The utilization of such platforms will significantly reduce the TAT for antibiotic susceptibility determination, thus enabling the selection of enhanced, target-specific therapies [15]. Diagnostic tests are considered an essential weapon in any strategy against AMR. Rapid diagnostic tests (RDTs) related to infectious diseases are considered an indispensable tool for antimicrobial stewardship programs. RDTs have shown to reduce mortality, lessen hospital stay, and shrink healthcare costs. Indeed, such diagnostic tests have proven to be more cost-effective, not only by providing a significant cost reduction, but also by decreasing antibiotic use [17,18,19,20].

**Figure 1:**
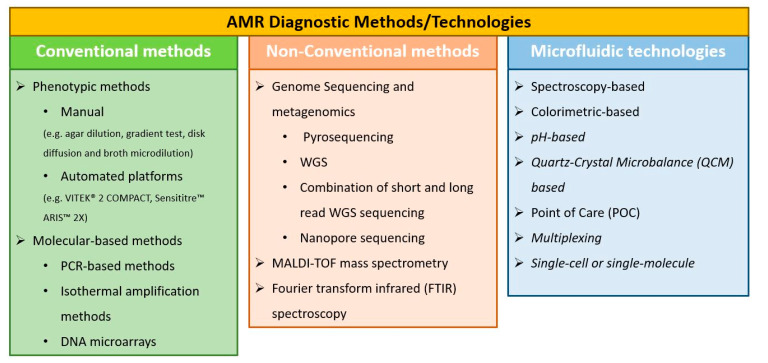
Summarizing chart of the methods and technologies analyzed in the present review.

The present review aims to give an outline of the current and emerging methods and technologies being implemented, or under development, targeting fast detection of antimicrobial resistance. Moreover, the main advantages and limitations of these methods and technologies are summarized. Already established methods, such as phenotypic and molecular-based techniques, as well as the more recently developed sequencing (whole genome sequencing (WGS) and whole genome metasequencing (WGM), MALDI-TOF MS, and IR spectroscopy, are also subjected to a critical overview. A special focus is placed on those state-of-the-art approaches, such as microfluidics and lab-on-a-chip technologies, which have a promising potential in AMR detection. Finally, we provide a short summary of the commercially available platforms designed for AST. Figure 1 depicts a summarizing chart of the methods and technologies analyzed in the present review.

## 2. Conventional AMR Diagnostic Methods

Although new time-saving technologies have been introduced to obtain antimicrobial resistance data, the classic, conventional technologies are still being used. These mainly include culture-based and molecular-based approaches. More recently, microscopy-based and spectrometry-based approaches have also been incorporated in the tools for developing diagnostics.

### 2.1. Phenotypic Methods

Culture-based methods rely on the phenotypic resistance detection by evaluating the bacterial growth in the presence of antibiotics, and can be classified in two categories, manual and automated. Manual tests include agar dilution, gradient test, disk diffusion, and broth microdilution antimicrobial susceptibility testing methods. The automated commercial platforms (VITEK® 2 COMPACT, Sensititre™ ARIS™ 2X, and Alfred 60AST system) use some of the aforementioned methods. Broth dilution-based platforms typically use ready-made cartridges or plates including positive controls and gradient concentrations of antibiotics. Sensititre panels belong to the category of microdilution methods. Typically, such panels are plastic multi-well micro-titer plates precision-dosed with dried antimicrobial agents. For instance, the Sensititre panel method was used for the determination of the susceptibility of carbapenem-resistant *Klebsiella pneumoniae* to polymyxins [21]. Such platforms usually offer real-time growth monitoring and minimum inhibitory concentration (MIC) analysis through their comprehensive databases which include a broad spectrum of organisms. The above-mentioned technologies offer qualitative and quantitative data for the strain under investigation. For example, dilution methods and Epsilometer tests (E-tests) provide quantitative values [22] for the minimum inhibitory concentration (MIC), as the lowest concentration of a given antimicrobial which prevents the visible overnight growth of a culture [23]. Disk diffusion provides a zone of inhibition. E-Test belongs to the gradient test methods [24] and is especially useful for fastidious microorganisms [25], such as *Campylobacter* spp. [26]. Various methods have been traditionally employed regarding the phenotypic analysis for susceptibility of bacteria to antibiotics, and different standards, criteria, and guidelines have been proposed by several international organizations for the interpretation of Alfred 60 antimicrobial susceptibility testing (AST) results. The European Committee on Antimicrobial Susceptibility Testing (EUCAST) and the Clinical and Laboratory Standards Institute (CLSI) in the USA are two of the main organizations responsible for the annual revision and update of the AST standards. However, several discrepancies have been observed in the interpretation of the criteria regarding different bacterial species. For example, in the case of amikacin resistant *Escherichia coli*, a more stringent susceptibility breakpoint is provided by EUCAST (≤8 mg/L) compared to CLSI (≤16 mg/L) [22].

### 2.2. Molecular-Based Methods

Molecular-based assays addressing the detection of ARG can offer advantages over phenotypic assays, such as multiplex targeting and more precise characterization and detection of AMR genes. For some taxonomic units, susceptibility breakpoints have not been established, and molecular-based methods represent an acceptable alternative. Another advantage is the elimination of isolate purification since non-purified polymicrobial samples can be used. Moreover, they allow for relatively quick adaptation to newly introduced resistance factors [27]. Nevertheless, molecular-based assays for AMR detection have some limitations. Molecular-based methods are not capable of defining MIC. Besides, some ARGs could be missed in terms of both sensitivity and coverage since they can only detect resistances that are searched for and not newly evolved ones. Moreover, the wide diversity of different genes related to AMR poses a challenge in assay development due to the cost involved, thus competing with phenotypic assays is sometimes difficult. However, advancements in the field of molecular based techniques are gaining a place in routine diagnostics [28]. Molecular-based methods for detecting ARGs as well as their expression take advantage of the developments in amplification and nucleic acid hybridization techniques [29]. Molecular-based techniques can offer ARGs detection in a fast and sensitive manner. ARGs encode the ability of bacteria to survive and grow in the presence of antibiotics. In the past, scientists were solely targeting a small fraction of ARGs, but with the decrease in the cost of next-generation sequencing (NGS) technologies and the subsequent expansion in bacterial whole genome sequencing (WGS), the availability of ARG targets in various databases has enormously been expanded [30]. In the following sections, nucleic acid amplification-based techniques, such as polymerase chain reaction (PCR) and isothermal techniques, as well as DNA microarrays, will be discussed. 

#### 2.2.1. PCR-Based Methods

PCR is the most commonly used nucleic acid amplification technique for the detection of ARGs [31,32]. More recently, real-time [33], quantitative [34], digital [35,36], and multiplex [37] PCR assays have further boosted clinical acceptance of genetic testing.

The changes in NGS and WGS have impacted the availability of ARG targets, paving the way for high throughput quantitative PCR (HT-qPCR), which is comparatively fast, convenient, and allows for simultaneous investigation of a large number of ARGs [30]. HT-qPCR is cost effective and it has already been employed in many studies for the analysis of ARGs stemming from various sample types [38]. For example, Wang et al. used HT-qPCR to provide a comprehensive profiling of ARGs in bacteria isolated from park soils [39], whereas a novel high-throughput screening method (simultaneous screening 48 isolates against three antibiotics) employing HT-qPCR, tested the antimicrobial susceptibility of *Orientia tsutsugamushi* clinical isolates [40]. Xu et al. demonstrated the versatility of chemically synthesized double-stranded (ds) DNA, which can be employed as a qPCR standard for ARGs offering comparable performance, in terms of sensitivity and reliability, to natural DNA. This qPCR method has been successfully used with various sample types, such as animal feces, soil, and surface water [41]. A multiplex real-time PCR was used for AMR characterization in *Neisseria gonorrhoeae* including resistance to ciprofloxacin, ceftriaxone, cefixime, azithromycin, and spectinomycin. Although this methodology accurately detected mutations generating resistance to antibiotics employed for gonorrhea treatment, the low assay sensitivity prohibits the direct application for diagnostic testing in clinical specimens. Nevertheless, it can be used as a screening method for AMR in gonococcal isolates since it is faster than current conventional culture-based AMR testing [42]. Wang et al. developed a singleplex and a multiplex real-time PCR assays for methicillin resistant *S. aureus* (MRSA) in pediatric samples. The assay proved fast, reliable, and capable of detecting and differentiating MRSA and methicillin susceptible *S. aureus* MSSA [43]. Two decades ago, ligation mediated PCR (LM PCR) coupled with low denaturation temperature method has been proposed leading to specific melting-profile DNA patterns, both fungal and bacterial isolates. This method is suitable for strain characterization and differentiation [44]. This method has been used for epidemiological typing of various pathogens, such as extended-spectrum-beta-lactamase-producing *Escherichia coli* [45,46] as well as *Enterococcus faecium*, *Staphylococcus aureus*, *Klebsiella pneumoniae*, *Acinetobacter baumannii*, *Pseudomonas aeruginosa*, and *Enterobacter* [47].

#### 2.2.2. Isothermal Amplification Methods

A more recent development, in molecular biology, is the use of isothermal DNA amplification eliminating the need for thermocycling, which is indispensable in the case of traditional PCR methods. Several methods of isothermal nucleic acid amplification have been developed, such as strand displacement amplification (SDA), transcription mediated amplification (TMA), nucleic acid sequence-based amplification (NASBA), rolling circle amplification (RCA), recombinase polymerase amplification (RPA), loop-mediated isothermal amplification (LAMP), and helicase-dependent amplification (HDA) [48]. These methods have paved the way for the implementation of rapid, next-generation molecular diagnostics [49].

The main advantages of the isothermal over the conventional PCR-based methods are the circumvention of thermocycling, which in turns lead to low power consumption and reduced analysis time. Thermocyclers are no longer needed, since a water bath or a hotplate can regulate the temperature [50]. Moreover, unlike PCR, isothermal amplification is faster and more sensitive [51] since it does not depend on discrete thermal cycles, but rather relies on continuous amplification, which can yield traceable amplicons in less than 10 min. Another advantage offered by some isothermal methods, such as LAMP, RCA, and HDA is the elimination of template denaturation and the tolerance to biological components for LAMP and HDA [52]. Moreover, although some isothermal methods have complex primer design (e.g., LAMP) they offer greater specificity compared to PCR. A recent evaluation of several isothermal methods in terms of simplicity, sensitivity, cost, and reproducibility showed that LAMP and RPA hold great potential for point-of-need (PON) diagnostics employed in low resource settings. Both of them are single step (incubation at a single temperature) and require minimum amount of DNA template [50]. Isothermal methods are also preferable for microfluidic-based approaches due to all of the aforementioned reasons [53]. In addition, LAMP amplicons can be detected even with naked-eye through turbidity or color change [54]. On the other hand, isothermal methods also have some limitations. Multiplexing approaches of isothermal methods are less successful, since the difficulty of the experimental design is increased [55]. Furthermore, some isothermal amplification methods have complex reaction mechanisms and need several primers, for example LAMP needs 4–6 primers, or several enzymatic steps are involved, such as in NASBA [52].

During the last two decades, significant investments in engineering, reagent formulations, and software have resulted in the commercialization of in vitro diagnostic (IVD) products based on PCR and isothermal nucleic acid amplification technology (NAAT) [56]. The integration and automation of processes, such as nucleic acid extraction, purification, amplification, and detection, coupled with sophisticated data analysis software have led to integrated and automated platforms (discussed in subsequent sections of this review article) providing accurate results [57].

#### 2.2.3. DNA Microarrays

A DNA microarray is a tool, which allows for the assessment of the bacterial genomic diversity. This approach relies on the detection of the presence or absence of genes in a target organism when compared to a reference strain or genome. Initially, DNA microarrays were based on glass slides [58], which were spotted with numerous specific DNA probes relying on reference genes present in a characterized strain for which the whole-genome sequence was available. Comparative genomic hybridizations were performed followed by the analysis of the hybridization results. However, the use of glass slides as well as fluorescent dyes made the process costly and time-consuming. Nonetheless, there have been numerous advancements in the DNA microarray technology during the past two decades [59]. A fast and simple DNA labeling system based on biotinylated primers specific for the linkers has been developed for disposable microarrays [60]. A DNA microarray for the simultaneous (multiplex asymmetric PCR amplification) detection of ARGs among *Staphylococcus* clinical isolates based on fluorescently labeled PCR products has been developed [61]. More recently, Havlicek et al. proposed a rapid cartridge based, melting curve assay for the detection of pyrazinamide resistant *Mycobacterium tuberculosis* [62]. The assay can be automatically implemented using a closed cartridge coupled with a battery powered Alere™ q analyzer, as a point-of-care test in resource-limited settings [62].

## 3. Non-Conventional AST Methods

In this section, some of the most promising non-conventional methods for AST will be described. Those method include: sequencing, matrix-assisted laser desorption/ionization time-of-flight mass spectrometry (MALDI-TOF MS), and Fourier transform infrared (FTIR) spectroscopy.

### 3.1. Genome Sequencing and Metagenomics in AMR Diagnostics

The first DNA sequencing methods were developed in the mid-1970s and were able to decode hundreds of nucleotide bases of DNA per day. At that time, the two most widely accepted methods were the chain terminator [63] and the chemical cleavage procedures [64]. Single-base resolution was enabled by polyacrylamide gel electrophoresis for each base-specific reaction. In 1995, the first complete bacterial genome (*Haemophilus influenzae*, 1,830,137 bp) was obtained with the first automated sequencers employing fluorescence chemistry based on the Sanger method [65]. Until 2005, the Sanger sequencing prevailed as the primary sequencing technology. Although these first-generation sequencing methods had low throughput, they could produce high-quality, relatively long DNA sequences. Multiple sample sequencing was feasible by integrating numerous capillaries on the same instrument, thus enabling the sequencing of each individual sample. The major technical advancement of next-generation sequencing (NGS) was multiplexing, allowing for the simultaneous analysis of thousands of samples. Typically, a NGS workflow comprises DNA extraction and fragmentation, adaptors ligation, DNA amplification, and sequencing.

In second generation sequencing, or short-read sequencing, the template amplification encompasses intrinsic drawbacks, such as copying errors, sequence-dependent biases, and information loss. In 2005, the 454 pyrosequencing platform was introduced [66]. Pyrosequencing is based on the detection of pyrophosphate release along with the light generation on nucleotide incorporation, unlike the chain termination with dideoxynucleotides used in Sanger sequencing. The Illumina platforms, which use synthesis technology where reversible terminator nucleotides labeled with fluorescence are incorporated into DNA strands and visualized via their fluorophore excitation, were subsequently incorporated with the same aim [67].

On the other hand, the third-generation sequencing, first developed in 2011 by Pacific Biosciences, is a real-time and single molecule based long-read sequencing relying on an optical approach coupled with a zero-mode waveguide on a nanostructured device [68]. Oxford Nanopore Technologies developed another approach relying on DNA molecules movement through a nanopore and measuring an electrical signal changing analogously to the base presently passing the pore [69]. These newly introduced second and third generation sequencing approaches have paved the way for single genome sequencing, as well as for the characterization of complex microbial communities and the identification of antibiotic resistance determinants [70]. Whole metagenome sequencing (WMS) and analysis of genetic material in patient samples allows for the identification of ARG directly from clinical specimens without the need for prior isolation or identification of specific bacteria.

Bacterial sequence data availability has increased due to the advancements in sequencing technologies. Improved computational methods coupled with the continual cost decrease (due to the intense competition among different companies) made sequencing an affordable and viable tool for ARG identification, characterization, and surveillance [71]. Numerous methods, tools, and databases (Table 1) have been reported in recent years for the detection of genetic determinants related to AMR from WGS [72] and WMS data [73]. These evolving methods and technologies act as complementary tools to traditional culture-based methods, providing opportunities for rapid and sensitive resistance determination in uncultivable and cultivable bacteria. More information on the use of databases for AMR detection can be found in two recent reviews [74,75]. The organization of sequencing data is considered a crucial pre-processing step prior to ARG analysis. Short reads, produced by technologies like Illumina, could be processed employing assembly-based methods (sequencing reads are initially assembled into contiguous fragments (contigs) followed by annotation where comparison takes place with public or custom reference databases), or directly analyzed utilizing read-based methods where resistance determinants are forecasted by mapping reads to a reference database [75].

A main advancement facilitating resistome surveillance is the established power for AMR prediction from solely genomic data. Various studies along with those focused on foodborne pathogens have demonstrated a high (>96%) concordance between the presence of known mutations or ARGs and MIC of various antimicrobials [76,77,78,79]. In addition, a growing body of evidence shows that it is feasible to predict AMR and sometimes also the MIC of an antimicrobial, by employing machine learning techniques to genome sequencing data [80,81].

Although long-read sequencing platforms can provide comprehensive entire genome capturing, they require substantial investment not only in equipment, but also in laboratory expertise. In addition, such systems typically require substantial quantities of DNA (i.e., more than 5 μg) and longer preparatory time, and they have higher error rates as compared to short-read sequencing platforms. Alternative sequencing platforms relying on nanopore technology are considered capable of providing libraries of high quality from long reads, as well as producing closed bacterial genomes. In addition, advantages, such as portability and affordability, less laboratory space, and on-site sequencing, have been highlighted [82]. The MinION nanopore system (Oxford Nanopore, Oxford, UK), is a portable (palm-sized, 100 g), real-time device for DNA and RNA sequencing, able to detect changes in ionic current upon DNA or RNA passing through the nanopores.

#### 3.1.1. Pyrosequencing

In 2012, pyrosequencing was suggested as an innovative, rapid tool for the detection of *Yersinia pestis* strains towards fighting bioterrorism. The detection and identification relied on virulence genes, which led to an assay based on pyrosequencing for characterizing ARG profiles was developed by Amoako et al. [83]. Pyrosequencing was also evaluated as a tool for the detection of clinical drug-resistant *Mycobacterium tuberculosis*. The pyrosequencing assay was capable of reliably and robustly detecting resistance-associated mutations in *M. tuberculosis* isolates with great specificity (96–100%) [84]. The efficiency of the pyrosequencing was evaluated based on the rapid detection of resistance to fluoroquinolones (FQs), rifampicin (RIF), kanamycin (KAN), and capreomycin (CAP) in *M. tuberculosis* clinical isolates [85]. The sensitivity of the assay for detecting the resistance to RIF, FQs, CAP, and KAN was 100%, 100%, 40%, and 50%, respectively, with 100% specificity. This assay was considered as a fast and effective method for the detection of mutations associated with drug resistance in *M. tuberculosis* clinical isolates [85]; however, it has been superseded by other sequencing technologies (see below).

#### 3.1.2. WGS

WGS for predicting AMR in non-typhoidal *Salmonella* was evaluated in human and food isolates employing v2 or v3 chemistry with paired-end 2- by 25- or 2- by 300-bp reads on the MiSeq platform (Illumina, San Diego, CA, USA) [86]. The data suggested that acquired resistance is highly correlated with the presence of known resistance determinants, useful for risk assessment linked to drug use in food animal production [86]. Velez et al. proposed the use of WGS for the determination of the occurrence of ARGs in *Streptococcus uberis* and *Streptococcus dysgalactiae* isolates, stemming from dairy cows [87]. A paired-end 125 bp sequencing was implemented using the Illumina HiSeq 2500 platform with v4 chemistry. In addition, they investigated the relation between genomic and epidemiological characteristics and phenotypic AMR profile. The outcome showed the association between a number of unique ARG sequences and phenotypic resistance (MIC data) [87]. Zhao et al. tried to identify AMR genotypes for *Campylobacter* investigating the correlation between resistance genotypes and phenotypes employing in vitro AST and WGS [88]. A strong correlation (99.2%) was observed between resistance phenotypes and genotypes. These outcomes suggested that WGS is a reliable resistance indicator (for tetracycline, ciprofloxacin, nalidixic acid, erythromycin, gentamicin, azithromycin, clindamycin, telithromycin, and florfenicol). From these initial screenings, several studies [74,89,90] also highlighted that WGS is a powerful tool for AMR surveillance programs [88]. More recently, an ongoing epidemiological change was studied using WGS revealing the co-existence of antibiotic resistance and virulence factors in carbapenem-resistant *Klebsiella pneumoniae* isolates, suggesting that this finding should be taken into account for future genomic surveillance studies [91].

#### 3.1.3. Combination of Short and Long Read WGS Sequencing

Plasmids are capable of transferring ARGs among bacterial isolates. Nonetheless, plasmids are difficult to assemble from short-read WGS data. Berbers et al. used short and long read WGS sequencing to characterize ARGs on plasmids as well as establishing their localization [92]. Due to the rising concern of the spread of ARGs, it is of crucial importance to establish their location, especially when they are in mobile elements. Risk assessment of AMR spread was feasible by overcoming the challenges of plasmid reconstruction when employing the combination of long and short read sequencing [92].

#### 3.1.4. Nanopore Sequencing

Nanopore sequencing has been widely used on viruses [93], yeasts [94] and for performing de novo bacterial assembly [95]. It has also been used for identifying viral pathogens [96], undertaking metagenomics studies [97] and detecting ARGs [98]. The MinION nanopore sequencer was implemented to resolve the structure as well as the chromosomal insertion site of a composite antibiotic resistance island in *Salmonella* Typhi [99]. It was also employed for the identification of the position as well as the structure of bacterial AMR determinants in a multidrug-resistant (MDR) strain of Enteroaggregative *E. coli* [100]. Long-read analysis of WGS data facilitated the identification of mobile genetic elements where AMR determinants were positioned and revealed the combination of various AMR determinants co-located on the same mobile element. These findings provided a deeper understanding regarding the transmission of co-located AMR determinants in MDR *E. coli* [100]. Schmidt et al. showed that MinION could successfully identify bacterial pathogens as well as acquired resistance genes without culturing directly from urine samples within 4 h [101]. This study highlights the importance of WMS-based diagnosis towards adjusting antimicrobial therapy [101]. The Oxford Nanopore MinION long read DNA sequencing device was exploited for the detection of ARGs, the assessment of ARGs’ taxonomic origin as well as to decoding their genetic organization and possible correlation with mobilization markers. Based on the findings, targeted intervention measures could be implemented in order to mitigate the risks of ARGs transferring among sites and, thus, improve biosecurity practices in hospitals and other environments [102]. Nanopore sequencing was also used for the fast determination of plasmids, virulence markers, phages and ARG in Shiga toxin-producing *E. coli* [82]. More recently, MinION nanopore sequencing was employed for rapid pathogen, plasmids and ARG identification in bacterial DNA extracted from positive blood cultures [103]. After only 10 min of sequencing, pathogen identification was possible. The detection of predefined ARGs and plasmids stemming from monoculture experiments was achieved within 1 h employing raw nanopore sequencing data. This is one crucial difference between Illumina and nanopore sequencing. Nanopore sequencing offers real-time data availability whereas when Illumina is used, the data become accessible once the sequencing run is finished [103]. The use of the MinION sequencer was also examined both for whole genome generation and characterization of *Streptococcus suis*. The genomes from the MinION sequencer were capable of accurately predicting the multilocus sequence type (8 out of 10 samples) and identifying AMR profiles (100% of the samples) [104]. The ultra-long read Nanopore sequencing technology was used for AMR detection in *Mannheimia haemolytica* [105]. De novo assembly generated a complete genome for a non-resistant and an almost complete assembly for a drug resistant strain. Successful ARG detection was achieved with only 5437 MinION reads [105].

Contrary to phenotypic tests, providing information solely related to AST, NGS, can reveal the molecular basis of the AMR resistance. The acquired information can be fed in monitoring schemes, aiding the understanding of the events leading to resistance acquisition. Furthermore, NGS is capable of characterizing novel mechanisms of resistance when they are detected. This can be achieved by sequencing isolates previously proven to be phenotypically resistant, thus providing an exquisite added value when compared to various nucleic-acid based techniques (e.g., PCR) [106].

### 3.2. MALDI-TOF Mass Spectrometry in AMR Diagnostics

Matrix-assisted laser desorption/ionization time-of-flight mass spectrometry (MALDI-TOF MS) can be used for the detection of AMR, alternatively to traditional genotypic or phenotypic bacterial characterization [107,108,109,110,111]. MALDI-TOF MS relies on the cellular proteome and is capable of profiling proteins (mainly ribosomal, 2–20 kD) from whole bacterial cell extracts creating a bacterial spectral fingerprint or profiles that discriminates microorganisms at a genus, species, and subspecies level [112,113].

In the assay preparation, the sample is mixed with a matrix, an energy absorbent solution. The entrapped sample within the matrix crystalizes upon drying. Once the sample is hit by a laser beam it gets ionized, producing protonated ions which are accelerated by using a constant potential leading to their separation based on their mass to charge (m/z) ratio. This ratio is determined by measuring the time needed for each protonated ion to move along the length of the tube. A “Peptide Mass Fingerprint” (PMF), which is a distinctive mass spectrum, is generated according to the TOF information. The peaks obtained from the PMF are compared to a database with reference peaks specific to genera and species of known, well characterized microorganisms, thus, allowing for the identification of the sample [114,115].

MALDI-TOF MS has also allowed the detection of antibiotic resistance mechanisms (e.g., carbapenemases) [116]. The standardization of the procedure is necessary to obtain reproducible results [117]. MALDI-TOF MS is considered a reliable, rapid (within minutes), accurate, easy to use, cost-effective, and environmentally friendly methodology [114]. Although MALDI-TOF MS has enormously decreased the TAT for bacterial identification and progress have been made towards the determination of AMR, the high cost (purchase and maintenance) as well as the large size of such systems pose significant restrictions for its implementation in low-resource settings or as a point-of-care (POC) AMR or AST testing platform [113]. Moreover, MALDI-TOF MS is not suitable for the characterization of mixed samples, since purification, cultivation as well as sample preparation procedures are required beforehand. Furthermore, additional chemicals, such as the matrix, are required for the execution of the tests [118]. Databases with spectra able to differentiate susceptible and resistant strains should be available. Table 2 includes recent works where MALDI-TOF MS has been applied to AMR detection. MALDI Biotyper (Bruker Daltonik Bremen, Germany), and VITEK MS (bioMérieux, Marcy l’Étoile, France) are the two commercially available MALDI-TOF MS systems. Comparison studies regarding the performance of both platforms can be found in the literature [119,120,121].

### 3.3. Fourier Transform Infrared (FTIR) Spectroscopy in AMR Diagnostics

Recently, great progress has been achieved in optical technologies and their applications in the biomedical and microbiology fields. Infrared (IR) spectroscopy and microscopy allows for enhanced spectral and spatial resolution facilitating the acquisition of biochemical information at molecular level for microorganisms. With respect to clinical microbiology applications, Fourier transform infrared (FTIR) spectroscopy is a phenotypic method that has emerged as an attractive and dynamic weapon enriching the tools employed for biochemical analysis, owing to the detailed information it can provide the chemical composition at molecular level. FTIR spectroscopy allows for the quantification of the IR light absorption by molecules such as lipids, lipopolysaccharides, carbohydrates, proteins, and nucleic acids, resulting in a characteristic FTIR spectrum that represents the complete composition of the sample [139]. These characteristic spectra of the cell biomolecules offer ample functional and structural information. IR spectroscopy has been applied to differentiate the molecular changes associated with the development of AMR in prokaryotes [140,141,142].

The coupling of IR spectroscopy of bacterial samples with data analysis employing artificial neural networks (ANNs) was able to detect uropathogenic *E. coli* strains susceptible to cephalothin, achieving a success rate of 95% [143]. In 2017, Sharaha et al. used FTIR to identify bacterial susceptibility to certain antibiotics based on the obtained IR bacterial spectra. An IR microscope was utilized, and a computational classification method was developed to analyze the IR spectra by novel pattern-recognition tools, to determine *E. coli* susceptibility to ceftazidime, gentamicin, nitrofurantoin, nalidixic acid, and ofloxacin. The results showed an 85% success rate in the classification into sensitive and resistant strains [144]. In 2017, Salman et al. demonstrated the detection of structural molecular changes linked AMR by employing FTIR microscopy coupled with a novel statistical classification approach developed in-house for spectral analysis [140]. Kochan et al. recently reported the identification of changes in the chemical composition of *S. aureus* associated with vancomycin and daptomycin antibiotic resistance. An innovative, single cell, nanoscale technique, namely atomic force microscopy-infrared spectroscopy (AFM-IR), coupled with chemometric analysis was employed [145]. AFM-IR combines IR and scanning probe microscopy to improve resolution and capacity to map cell structures at the atomic scale.

FTIR shows many advantages, such as reliability, speed, cost-effectiveness, and environmentally friendly methodology in AMR study. Similar to other instrumental systems (i.e., MALDI-TOF MS), the purchase and maintenance costs and equipment size make its implementation very difficult in low-resource settings, or as a POC AMR, or AST testing platform. Purification, cultivation, as well as sample preparation procedures are required previously, and databases with spectra able to differentiate susceptible and resistant strains should be available.

## 4. Microfluidics and Lab-on-a-Chip Technologies towards Rapid Diagnostics

Lab-on-a-chip (LoC) devices using microfluidics represent a promising tool in numerous fields, such as clinical diagnostics [146], food safety [147] and environmental monitoring [148]. Recently, LoC technology has also been applied in the detection of antibiotic-resistant bacteria [3]. Some of the advantages offered by the LoC technology compared to macro-scale methods are: fast and high throughput analysis, accurate fluid manipulation, low cost, low reagent, and power consumption, smaller sample volume, automation, integration, compactness, and portability [149,150,151,152]. Genotypic and phenotypic assays are the two main categories of microfluidic-based detection methods. Genotypic microfluidic assays (e.g. PCR, LAMP) target genetic markers (e.g., ARG), thus circumventing bacterial growth and allowing for shorter TAT (several hours) [153]. The implementation of microfluidics combined with isothermal DNA amplification protocols offer enhanced features due to the elimination of thermal cycling [50]. This approach is highly promising for the development of cheap, convenient, and efficient diagnostic tools for food safety, clinical, and environmental applications [154]. On the other hand, phenotypic microfluidic assays monitor bacterial growth of bacteria in the presence of antibiotics, thus offering accurate AST results. In them, in general, bacteria are confined in small volumes (e.g., chambers, channels, or droplets) [155], captured with the aid of antibodies on magnetic beads or membranes [156], or encapsulated in chambers containing agarose [157] and hydrodynamic trapping [158]. For example, hydrodynamic trapping is a method used for the immobilization of the bacteria and is compatible with microfluidics offering highly dense trap arrays, easy integration, high scalability, and easy biosensing though, the trapping efficiency is quite low. A drawback regarding the use of antibodies is the high cost as well as the restricted availability to specific strains. As for the droplet-based method, typically they require expensive and sophisticated readout. The agarose-based method, although it can be applied to conventional multi-well plates, the arraying is not straightforward, which hinders both the automated detection and the data analysis. Due to these limitations, more research and improvements are needed in order for these systems to become commercially available. In the following sections, various approaches will be discussed; namely spectroscopy-based, colorimetric-based, pH-based, and, last but not least, quartz-crystal microbalance (QCM) based, point-of-care (POC), multiplexing, single-cell, or single-molecule.

### 4.1. Spectroscopy-Based Approaches

Surface enhanced Raman spectroscopy (SERS) is considered a main biochemical fingerprinting approach since it can precisely reflect the macromolecular profiles as well as the changes occurring within the bacterial cells as a result of antibiotic action [118,159]. SERS has been applied for the investigation of the resistance or susceptibility to antibiotics of bacteria, as well as for studying the working mechanism of antibiotics relying on the whole cells’ spectral fingerprint. SERS is capable of providing rapid, accurate, and ultra-sensitive detection of resistant bacteria with minimum requirement for sample preparation and handling [118,160]. SERS has also been used in LoC platforms. Lu et al. reported the development of a microfluidic chip combined with SERS providing rapid detection and differentiation of MSSA and MRSA [161]. Chang et al. presented the development of an integrated multimodal microfluidic system capable of performing on-chip enrichment of bacteria, collection of metabolites, and in situ SERS measurements for AST, with a limit of detection (LoD) of 10^3^ CFU/mL [162]. Liao et al. reported the development of a microfluidic platform integrating SERS with microwells allowing for low concentration (10^3^ CFU/mL) encapsulation of bacteria followed by label-free detection and in situ AST [163].

However, while current advancements in SERS methodology have substantially improved the selectivity and sensitivity in bacterial biosensing, it still has some limitations. It usually requires a drying step of the sample prior to analysis that can lead to reproducibility issues. Although liquid phase detection of bacteria is favored when interrogation of cells is performed under their natural environment, this is frequently challenging because of scattering of the Raman laser source. Another limitation associated with SERS is the sample and the experimental conditions, i.e., typically, samples containing a single bacterial species are employed under regulated laboratory conditions. Furthermore, despite the progress made in the identification of the molecular spectral fingerprints (e.g. nucleobases), comprehensive databases of SERS spectra of biomolecules are still needed, as well as mathematical interpretation and processing of spectra (e.g., multivariate data analysis) [164]. Ideally, bacterial SERS biosensors should facilitate the simultaneous detection of multiple strains from complex samples. Further information on the SERS method can be found in the review of Galvan et al. [159]. 

### 4.2. Colorimetric-Based Approaches

Several studies also report the development of colorimetric-based microfluidic platforms addressing pathogen identification and AST. Lee et al. proposed an integrated, automated, microfluidic platform capable of performing AST for 1–2 antibiotic combinations against bacterial pathogens [165]. On-chip determination of MIC is also provided via a colorimetric assay using a pH-dependent colorimetric broth. The total TAT of the on-chip microfluidic assay is 16–24 h, approximately. Automated fluidic control (e.g., transportation, mixing) is achieved using a pneumatically controlled custom-made module connected to the microfluidic chip. The initial loading of all samples [250 μL of bacterial suspension (10^6^ CFU/mL) /chamber] and reagents is performed manually [165]. Recently, Ma et al. proposed a polymer-based microfluidic device addressing the identification and AST of *Campylobacter* spp. The microdevice consisted of an array of incubation micro-chambers loaded with chromogenic medium and antibiotics. Bacterial growth was visualized through a color change (chromogenic reaction). Rapid and reliable on-chip identification and AST was performed within 24 h with a LoD of 10^2^ CFU/mL. Some variations in terms of the TAT and the LoD were observed according to the food matrix used [166].

### 4.3. pH-Based Approaches

Tang et al. proposed a microfluidic device integrating polymer-based microfluidic channels with a pH-sensitive chitosan hydrogel capable of detecting small pH changes for rapid AST [167]. Fourier transform reflectometric interference spectroscopy (FT-RIFS) was used for the real-time observation of the changes in the pH. The TAT for detection of whole bacterial growth was less than 2 h [167]. Hu et al. developed a real-time, ultra-fast electronic detection microdevice for ARG detection (resistance genes from *E. coli* and *Klebsiella pneumoniae*) using the RPA method for isothermal amplification coupled with a thin film transistor sensor for measuring changes in the pH. The TAT was less than 3 min for a LoD of 100 copies [168]. Xu et al. presented a polymer/paper hybrid microfluidic chip for a one-step identification and AST of multiple uropathogens. The multiplexed colorimetric assay was facilitated via the use of paper substrates within the cell culture microchambers, allowing for a versatile combination of the antimicrobial agents and the chromogenic media. The assay was completed within 15 h and the outcome of the chromogenic reaction was monitored via a camera. Snapshots were taken every 30 min and analyzed with an image analysis software [169]. Recently, He et al. reported a laser-pattern paper-based microfluidic device capable of performing *E. coli* identification and susceptibility testing via visual observation of a simple color change (colorimetric readout). Such a micro-device is suitable for low resource settings and can be used by minimally trained personnel [170].

### 4.4. Quartz-Crystal Microbalance (QCM)-Based Approaches

Quartz-crystal microbalance (QCM) is a physical nanogram-sensitive device with a piezoelectric sensor. QCM facilitates the real-time, rapid, on-site detection of AMR bacteria [171]. Reyes et al. have demonstrated a highly sensitive, accurate, and dynamic (real-time) system with a dual purpose, allowing both for monitoring of antimicrobial effects on *E. coli* and *Saccharomyces cerevisiae*, as well as ARG detection employing a magnesium zinc oxide (MZO) nanostructure-modified quartz crystal microbalance (MZOnano-QCM) biosensor [172]. Low cost, low demand in clinical samples volume, and rapidity (within 10 min) are the main advantages of the proposed method [173].

### 4.5. POC Approaches

Toosky et al. developed a POC system for AMR diagnostics and phenotypic AST addressing bacteriuria and urinary tract infection (UTI) [174]. The TAT is 2h with the ability of detection and quantification of bacterial concentrations ranging from 50 to 10^5^ CFU/mL. The detection is based on a portable particle-counting instrument comprising a miniature confocal microscope coupled with a software for real-time data analysis. The detection system allows for growth curve measurements of fluorescently stained bacterial cells in control and antibiotic-treated samples. The main advantages of the proposed POC lie in the elimination of pre-processing steps (e.g., pre-culture, enrichment, centrifugation) of urine samples as well as in the sensitivity of the instrument [174]. Only preliminary data are available for this method; thus, further studies are needed. One limitation of this method, which is common in all AST methods, is the negative effect of mixed cultures both on the specificity and the sensitivity of the results. Recently, Abram et al. reported a RDT platform integrating a novel single step blood droplet digital PCR assay with a high throughput three-dimensional (3D) particle counter system capable of performing bacterial identification and AST directly from whole blood samples, eliminating the need of culture and sample processing steps [175]. The demonstrated technology could simultaneously achieve high sensitivity of 10 CFU/mL and fast TAT of one hour [175].

### 4.6. Multiplex Approaches

A multiplex (eight samples) microfluidic chip for high throughput rapid phenotypic AST was proposed [176]. A mix of bacterial isolates and agarose was loaded in an array of microchambers within the chip. The growth rate of bacterial colonies under antibiotic gradients is monitored with the aid of a custom-built dark-field microscope coupled to a motorized camera (taking snapshots every 10 min) followed by automated image analysis. The TAT is 5 h and the method achieves stable MIC values showing 100% agreement with reference (broth microdilution) MIC values. The key advantage of the proposed system is the ability of simultaneously and rapidly analyzing eight samples on a single chip, which can also allow for parallel testing of several antibiotics [176].

### 4.7. Single-Cell or Single-Molecule Approaches

A rapid AST system based on a microfluidic agarose channel with immobilized bacteria allows for single cell growth and monitoring by microscopy [177]. MIC values were determined by analyzing the time lapse images of the single cell bacteria cultured under various antibiotic concentrations. The TAT for the aforementioned system was less than 4 h [177]. Baltekin et al. presented their rapid AST system, also based on single-cell imaging (phase contrast microscope), using a microfluidic chip (made of a micromolded silicon elastomer and a cover glass) with cell traps. The rapid AST system was used for the determination of urinary tract infections (UTIs) caused by resistant bacteria with a TAT of 30 min even when urine samples with low CFUs were used [178]. Li et al. reported a versatile microfluidic system for fast bacterial classification (3 min) and phenotypic AST at the single-cell level. The incorporation of tunable microfluidic valves coupled with real-time visual detection (microscopy) facilitated the cell entrapment and classification based on their size and shape. The TAT for determining susceptibility, by monitoring the growth of the bacteria (single-cell level) in the presence of antibiotics, was 30 min. Moreover, the proposed system can be extensively applied for bacteria detection and complex (blood cultures, urine, whole blood) polymicrobial samples analysis [179]. Table 3 summarizes the microfluidic platforms that have been described in the literature, together with their main characteristics.

## 5. Overview of Commercially Available AST Platforms

In this section, a description of some common, commercially available systems for AST follows.

Adagio™ Antimicrobial Susceptibility Testing System (Bio-Rad Laboratories) [180] is an automated system built around an imaging device. It measures the size of the inhibition zone around antibiotic discs. It is coupled with a sophisticated data management software allowing for rapid and accurate result generation and automated AST interpretation [181]. The Adagio system was evaluated for the automated reading and interpretation of disk diffusion AST results in bacteria. Good categorical agreement was observed after visual validation of the automated results [182].

VITEK® 2 COMPACT (bioMérieux, Marcy l’Étoile, France) is a compact, automated instrument addressing microbial identification and AST by reducing hands-on time for enhanced workflow and rapid reporting. The TAT is 2 to 18 h, although primary organism isolation is required. VITEK® 2 COMPACT is considered a cost-effective, space-saving system. The technology used by VITEK® 2 COMPACT relies on a fluorogenic methodology for organism identification and a turbidimetric method for AST.

Accelerate Pheno™ (Accelerated Diagnostics, USA) comprises a fully automated system capable of performing identification in approximately 2 h and AST within approximately 7 h directly from the sample without requiring culturing for isolates [183]. The clean-up process of the samples relies on gel electrofiltration. Pathogen detection, species identification, and quantitation are performed in a fast and fully automated manner using fluorescence in situ hybridization. It also incorporates an automated digital microscope for the morphokinetic cellular analysis (MCA), thus allowing tracking phenotypic features, such as size, shape, division rate of individual live cells, while being challenged by antibiotics, as well as extrapolating MIC values. The main advantage of this system is the user-friendliness, whereas the main disadvantages are the lack of freedom for any intervention and the necessity of processing solely fresh blood cultures.

Alfred 60AST system (Alifax, S.r.l., Italy) is a fully automated system capable of performing bacterial culture, residual antimicrobial activity (RAA) and susceptibility testing including the processes of sample inoculation, reading, and result transmission. This system, which relies on light scattering, is capable of detecting not only the presence of live bacteria, providing real-time information on growth curves as well as bacterial counts, but also their drug resistance in a few hours (4–6 h) with high sensitivity and specificity. The Alfred 60AST system coupled with MALDI-TOF MS for direct identification is considered a rapid AST. The main advantage of this system is its plasticity, since it allows for interventions by the user, which could also be considered as its main drawback, since such interventions dictate the need of skilled personnel able to interpret the results (i.e., growth curves).

MicroScan WalkAway plus System (Beckman Coulter, Inc.) (40 or 96-panel capacity models) provides identification of microorganisms and AST results efficiently with minimal labor in an automated manner from an isolate inoculum within 4 h (or overnight for some samples).

BD Phoenix™ (Becton, Dickinson, and Company) is an AST system providing rapid, reliable and accurate results from colony inoculums. It employs an oxidation/reduction indicator and a turbidimetric growth detector. Moreover, 200 identifications (ID)/AST sets could be processed in less than 4.5 h.

Sensititre™ ARIS™ 2X (Thermo Fisher) provides bacterial pathogen identification and emerging antibiotic resistance detection relying on the gold-standard of broth microdilution coupled with the time-saving advantages of automation, thus improving patient care and enhancing lab efficiency. Growth measurements and endpoint MIC determinations are based on the hydrolysis of a fluorogenic substrate by the bacterial isolates.

GeneFluidics (GeneFluidics, Inc.) offers automated platforms for research use, addressing both identification and AST. More specifically, ProMax®, UtiMax®, and BsiMax® platforms are capable of providing identification (TAT: n/a, 1 h, and 6 h, respectively) and AST (TAT: 3 h, 2 h, and 3.5 h, respectively) results from isolates, urine, and whole blood samples, respectively. GeneFluidics’ products rely on molecular-based, PCR-less identification of species-specific phenotypic markers of resistance and susceptibility (resistance profiling determined by the change in 16S rRNA content of each target pathogen under various antibiotics conditions). The detection technology relies on an electrochemical sensor array.

### Comparison of Platforms

Alfred 60AST coupled with MALDI-TOF MS has a faster TAT for identification and AST and it is more cost-effective compared to Accelerate Pheno™. However, Accelerate Pheno™ can provide identification and MIC determination using a single cartridge. Thus, it is considered an excellent candidate for small and medium laboratories, where MALDI-TOF MS equipment is not available [17]. From the above-mentioned platforms, VITEK2, BD Phoenix, MicroScan WalkAway and Sensititre ARIS 2X are cleared by the Food and Drug Administration (FDA) as IVD diagnostics. Although these platforms generate fast (2–18 h) results, it must be highlighted that a standardized microbial inoculum is required, which entails a culturing step of the specimen for 1–2 days prior introducing the inoculum into the AST platform [184]. Table 4 summarizes some of the most commonly used commercially available platforms for AST.

## 6. Conclusions and Future Perspectives

The AMR crisis is imposing a joint response from academia, risk managers, risk assessors, government, and industry to enhance the current methodologies, both for diagnosis and treatment, by developing novel tools circumventing the drawbacks and limitations of the golden standards and existing AST methods. The main limitations of the currently available tools are: (i) the need for sample pre-treatment steps; (ii) their low sensitivity; (iii) the incapacity of microorganism identification in some occasions; and (iv) the lack of integration, automation, and portability. In relation to the three first points, lengthy biological protocols (culturing, isolation, identification) are required in order to detect a number of pathogens. It is highly important to focus on and strive for substantial advancement towards the development of new testing platforms with superior performance characteristics in this regard, in order to allow for their approval and marketing as soon as possible. Spending time and effort on improvements on existing methods, technologies, and platforms is also plausible.

According to MarketsandMarkets™, by 2025, the AST market is projected to reach USD $4.2 billion. In this market report, it is highlighted that, despite the use of automated AST platforms reducing both the incubation and detection times, the high prices of these platforms are considerable constraining factors for the widespread adoption of such platforms by end-users, principally for small-budget institutions [192]. In terms of product type, manual AST products held the largest share of the overall AST market in 2019. This is mainly attributed to the lower cost of such products. Based on the method, in 2019, disk diffusion accounted for the biggest share of the AST market mainly attributed to the relatively low cost and the diversity of the commercially available disks. Regarding the end users, hospitals and diagnostic laboratories commanded the largest share of the AST market in 2019 [193]. The cost estimation of the methods and technologies reviewed in this paper is out of scope. Albeit some rough estimations on the cost related to AST methods is described in the following publications of El-Bouri et al, Vrioni et al. and Vasala et al. [26,114,194].

All of the methods and technologies described above have shown great potential towards the AMR challenge, though various issues remain unanswered. For example, how many of these methods are generally applicable? Have these methodologies been validated against reference methods? For those not commercialized yet, when is it anticipated to become commercially available on the market for broad use? Although many methods presented in the literature claim to be capable of performing AMR detection in a short amount of time (minutes–few hours), in reality, they do not consider tedious pre-treatment steps, such as culture enrichment and culture isolation.

To sum up, standard cultivation tests for AST typically have a TAT of 18–36 h and can provide MIC, though they are not suitable for non-culturable pathogens. The commercially available automated platforms have a TAT of 2–24 h, some of them provide MIC, but they are not compatible with non-culturable pathogens. MALDI-TOF MS has a lower TAT of 2–4 h and, in some cases, MIC determination is also possible, though it shares the same limitation as the two previous technologies. In addition, it is not yet endowed with standardized AST protocols as well as companion software for data analysis. NAAT-based approaches have a TAT of 0.5–4 h, though MIC determination is not possible. On the other hand, NAAT is suitable for AST for non-culturable pathogens. In addition, NAAT-based systems are capable of easily integrating the detection of emerging ARGs or mutations. Nevertheless, new validations and standardization are needed for the diagnostics for each update. As for the WGS, it is still relatively newly introduced in the field of rapid AST. The biggest challenge related to WGS is the bioinformatics, since universal databases are required for the result interpretation. Microfluidics is an ever-growing field with great potential and versatility. Various microfluidic technologies, coupled with miniaturized biosensing schemes, hold a great promise for the future. Such microfluidic devices offer many advantages over conventional platforms, such as minimal resource (sample, reagents, power) use, low cost, user-friendly handling, rapid TAT, integration (multimodal), automation, and portability. Regarding the microfluidic approaches, apart from the upscaling of the fabrication processes to allow for mass production at a low price [195], a high degree of integration is needed for the pretreatment steps (e.g., sample preparation) and user-friendly interfacing, so as to become more appealing to users.

Although all of these technologies struggle to meet the requirements for rapid AST, none of them is optimal. It is highly probable that some of them will claim a large share of rapid AST diagnostics market in the future. This market can be divided into two categories, the central lab-based and the PON-based. The first refers to organized and well-equipped labs (e.g., hospitals, research, and diagnostic centers) where WGS, WGM, PCR, MALDI-TOF MS, FTIR, and automated AST platforms can also be integrated, and the latter would be useful for small laboratories, practitioners, and pharmacists where microfluidic-based, portable AST platforms would be more appropriate, as they are superior in terms of portability and affordability, needing less laboratory space, and providing fast TAT at the same time. Table 5 summarizes the main advantages and disadvantages of the methods and technologies described.

In conclusion, the development of reliable, sensitive and affordable diagnostics will facilitate combating the threat of AMR. Rapid diagnostic technologies employed mainly in primary care locations (i.e., rapid diagnostic tests), could enhance and facilitate the effective and targeted treatment. Moreover, advanced monitoring systems, such as mobile applications, coupled with surveillance programs, are essential to track antimicrobial consumption. Emerging approaches, such as machine learning and data mining in combination with automation, will play a key role for the next generation diagnostics. Epidemiological surveillance is of upmost importance for AMR since it provides the necessary input for developing and monitoring therapy guidelines, antibiotic stewardship programs, public health interventions, and novel antimicrobials and vaccines [196]. The developments on the cutting-edge methods and technologies addressing AMR and AST, coupled with the surveillance programs allowing for increased and simplified data transmission, would hugely contribute towards minimizing the detrimental effects of the AMR threat.

## Figures and Tables

**Table 1:** Bioinformatic tools and databases for antimicrobial resistant gene (ARG) detection from whole genome sequencing (WGS) or whole metagenome sequencing (WMS) data.

Name	Type of Tool	Link
RGI	Assembly-based	https://card.mcmaster.ca/analyze/rgi (accessed on 15 January 2021)
CARD	Assembly-based	https://card.mcmaster.ca/ (accessed on 15 January 2021)
ARGs-OAP (v2	Assembly-based	https://galaxyproject.org/use/args-oap/ (accessed on 15 January 2021)
ARIBA	Assembly-based	https://github.com/sanger-pathogens/ariba (accessed on 15 January 2021)
NCBI–AMRFinder	Assembly-based	https://www.ncbi.nlm.nih.gov/pathogens/antimicrobial-resistance/AMRFinder/ (accessed on 15 January 2021)
PointFinder	Assembly-based	https://cge.cbs.dtu.dk/services/ResFinder/ (accessed on 15 January 2021)
ShortBRED	Read-based	http://huttenhower.sph.harvard.edu/shortbred (accessed on 15 January 2021)
SEAR	Read-based	https://github.com/will-rowe/SEAR (accessed on 15 January 2021)
KmerResistance	Read-based	https://cge.cbs.dtu.dk/services/KmerResistance/ (accessed on 15 January 2021)
PATRIC	Read-based	www.patricbrc.org (accessed on 15 January 2021)
SSTAR	Read-based	https://github.com/tomdemanbio/Sequence-Search-Toolfor-Antimicrobial-Resistance-SSTAR (accessed on 15 January 2021)
DeepArgs	Read-based	https://bench.cs.vt.edu/deeparg (accessed on 15 January 2021)
GROOT	Read-based	https://github.com/will-rowe/groot (accessed on 15 January 2021)

**Table 2:** Applications of MALDI-TOF MS for specific antimicrobial resistance (AMR) detection.

Organism	Antibiotic	Year (Reference)
*E. coli*	Polymyxins	2018 [122]
*E. coli*	Colistin	2019 [123]
*E. coli* *Klebsiella pneumoniae*	Beta-lactams (ESBL-producing isolates)	2019 [124]
*Staphylococcus aureus* *S. intermedius* *S. pseudintermedius*	Novobiocin Polymyxin B Acriflavine	2019 [125]
*S. aureus*	Methicillin	2019 [126]
*Candida auris*	Echinocandins	2019 [127]
*K. pneumoniae* *Bacteroides fragilis* *S. aureus*	Carbapenems (carbapenemase-producing isolates) Methicillin	2019 [128]
*Enterobacteriaceae*	Carbapenems (carbapenemase-producing isolates)	2019 [129]
*Enterobacteriaceae*	Carbapenem	2019 [130]
*Pseudomonas aeruginosa*	Beta-lactams (MBL)	2019 [131]
*Enterococcus faecium*	Vancomycin	2019 [132]
*K. pneumoniae*	Carbapenems (carbapenemase-producing isolates)	2019 [133]
*Acinetobacter baumannii*	Colistin	2020 [134]
*E. coli* *K. pneumoniae*	Cefotaxime Meropenem, Ciprofloxacin	2020 [135]
*S. aureus* *Enterococcus* species *E. coli* *K. pneumoniae*	Oxacillin (methicillin) Vancomycin Ceftriaxone Meropenem	2020 [136]
*Enterobacterales*	Imipenem/Relebactam	2020 [137]
*S. aureus*	Methicillin	2020 [138]

**Table 3:** Microfluidic platforms described in the literature.

Category	Method	LoD ^1^	TAT ^2^	Reference
Microfluidic	Optical (laser)	20-25 cells	3-5 h	[15]
Microfluidic	Colorimetric	100 CFU/mL	24 h	[166]
Microfluidic	Colorimetric	N/A ^3^	24 h	[165]
Microfluidic	Colorimetric	N/A	15 h	[169]
Microfluidic	Colorimetric	N/A	Overnight	[170]
Microfluidic	Microscopy	N/A	4 h	[177]
Microfluidic	Microscopy	N/A	30 min	[178]
Microfluidic	Microscopy	N/A	33 min	[179]
Microfluidic	Microscopy	N/A	5 h	[176]
Cuvette	Microscopy	50 CFU/mL	2 h	[174]
Microfluidic	Electrochemical (pH)	100 cells	3 min	[168]
Microfluidic	FT-RIFS	N/A	2 h	[167]
Microfluidic	QCM	N/A	10 min	[172]
Microfluidic	Digital PCR	10 CFU/mL	1 h	[175]
Microfluidic	SERS	10 ^3^ CFU/mL	N/A	[162]
Microfluidic	SERS	10 ^3^ CFU/mL	N/A	[163]

^1^ LoD: limit of detection, ^2^ TAT: turnaround time, ^3^ N/A: not applicable (this piece of information was not mentioned in the article).

**Table 4:** Commercially available AST platforms.

Name	Link	Detection Method	TAT ^1^	ID ^2^	AST ^3^	MIC ^4^	Reference
VITEK® 2 Compact (bioMérieux SA)	https://www.biomerieux-diagnostics.com/vitekr-2-compact-0 (accessed on 15 January 2021)	Turbidity	2–18 h	•	•		[185]
Adagio™ Antimicrobial Susceptibility Testing System (Bio-Rad Laboratories)	https://www.diagnostics-bio-rad.com/wp-content/uploads/2016/11/2015-Adagio-Brochure-EN.pdf (accessed on 15 January 2021)	Imaging device measuring the size of the inhibition zone around antibiotic discs			•	•	[180]
Accelerate Pheno™ (Accelerate Diagnostics, Inc.)	https://acceleratediagnostics.com/products/accelerate-pheno-system/ (accessed on 15 January 2021)	Fluorescence in-situ hybridization (FISH)	≈ 7	•	•	•	[186]
Alfred 60AST system (Alifax S.r.l.)	https://www.alifax.com/products/bacteriology-line/show/alfred-60 (accessed on 15 January 2021)	Light Scattering Technology/ Turbidity	4–6 h		•		[187]
ProMax ® (GeneFluidics, Inc.)	http://genefluidics.com/20151123/wp-content/uploads/2018/08/ProMax.pdf (accessed on 15 January 2021)	Electrochemical-based sensors based on sandwich hybridization of capture and detector probes with target 16S rRNA	3 h		•		
UtiMax® (GeneFluidics, Inc.)	http://genefluidics.com/20151123/wp-content/uploads/2018/08/UtiMax.pdf (accessed on 15 January 2021)	Electrochemical-based sensors based on sandwich hybridization of capture and detector probes with target 16S rRNA	3 h	•	•		[27]
BsiMax® (GeneFluidics, Inc.)	http://genefluidics.com/20151123/wp-content/uploads/2018/08/BsiMax.pdf (accessed on 15 January 2021)	Electrochemical-based sensors based on sandwich hybridization of capture and detector probes with target 16S rRNA	9.5 h	•	•		[188]
MicroScan WalkAway plus System (Beckman Coulter, Inc.)	https://www.beckmancoulter.com/es/products/microbiology/microscan-walkaway-plus-system (accessed on 15 January 2021)	Turbidity	4 h–overnight	•	•	•	[189]
BD Phoenix™ (Becton, Dickinson and Company)	https://www.bd.com/en-us/offerings/capabilities/microbiology-solutions/identification-and-susceptibility-testing/bd-phoenix-automated-identification-and-susceptibility-testing-system (accessed on 15 January 2021)	Turbidity and colorimetric change	4.5 h	•	•		[190]
Sensititre™ ARIS™ 2X (Thermo Scientific™)	https://www.fishersci.com/shop/products/sensititre-aris-2x-id-ast-inst/stv3090 (accessed on 15 January 2021)	Fluorescence measurement	Overnight (18 h–24 h)		•	•	[191]

^1^ TAT: turnaround time (TAT refers to both ID and AST when applicable), ^2^ ID: identification, ^3^ AST: antimicrobial Scheme, ^4^ MIC: minimal inhibitory concentration.

**Table 5:** Advantages and disadvantages of AMR diagnostic methods and technologies.

Method	Advantages	Disadvantages
**Conventional methods**		
Phenotypic methods	Reference, validated methods Simple methodology MIC ^1^ values can be estimated Usually, pathogen identification is also achieved	Testing of individual, purified strains Previous cultivation is needed (difficult for fastidious microorganisms, not possible for non-culturable ones) Some disagreements between standards For some taxonomic units, susceptibility cut-off values have not been yet established
Molecular-based assays	Elimination of sample purification Polymicrobial samples analyzed Multiplex targeting of AMR 2 determinants More precise detection and characterization of ARG 3 Relatively quick adaptation to newly introduced resistance factors	Need trained personal Expensive lab equipment Not capable of defining MIC Some ARGs could be missed (sensitivity and coverage) Diversity of ARG poses a difficulty in generating assays due to the cost involved Not total correlation with phenotype
**Non-conventional methods**		
WGS ^4^, WMS ^5^	Adequate for fastidious, non-culturable microorganisms For long-read sequencing platforms, portability and affordability, less laboratory space, and on-site sequencing Genetic basis of AMR established. Novel mechanisms of resistance can be characterized Simultaneous study of multiple AMR determinants (for WMS, from different hosts)	Large equipment costs Complex, laborious methodology Trained personnel needed Sometimes discrepancies with phenotypic tests (false positive, false negative results) For WMS, host of the AMR determinant is not known sometimes Not capable of defining MIC Not total correlation with phenotype
MALDI-TOF MS ^6^	Fast analysis High throughput Automated procedure Simple sample manipulation Low running costs Small sample volume Molecular basis of AMR established	Large equipment costs Testing of individual, purified strains. Previous cultivation is needed Databases (including spectra from resistant and susceptible strains) should be developed Need to find AMR biomarker (peak pattern). Not applicable to all microorganisms Mathematical discrimination procedure needed No portability Not capable of defining MIC
FT-IR ^7^ spectroscopy	Fast analysis High throughput Automated procedure Simple sample manipulation Low running costs Small sample volume	Large equipment costs Testing of individual, purified strains. Previous cultivation is needed Databases (including spectra from resistant and susceptible strains) should be developed Need to find AMR biomarker (spectral pattern). Not applicable to all microorganisms Mathematical discrimination procedure needed No portability IR ^8^ spectra influenced by culture conditions Not capable of defining MIC
**Technology**		
Microfluidics and Lab-on-a-chip (LoC ^9^)	Fast and high throughput analysis Accurate fluid manipulation Low cost, low reagent and power consumption Small sample volume Automated procedure Integration, compactness, portability Easy sample manipulation	Not capable of defining MIC Scalability issues Reproducibility issues in terms of fabrication Large surface to volume ratio Surface treatment (minimize adsorption) Commercialization

^1^ MIC: minimal inhibition concentration, ^2^ AMR: antimicrobial resistance, ^3^ ARG: antimicrobial resistance gene, ^4^ WGS: whole genome sequencing, ^5^ WMS: whole metagenome sequencing, ^6^ MALDI-TOF MS: matrix-assisted laser desorption/ionization- time of flight mass spectrometry, ^7^ FT-IR: Fourier-transform infrared spectroscopy, ^8^ IR: infrared, ^9^ LoC: lab-on-a-chip.

## Abbreviations

AFM-IR	Atomic Force Microscopy-Infrared Spectroscopy
AMR	Antimicrobial Resistance
ANN	Artificial Neural Networks
ARG	Antimicrobial Resistance Gene
AST	Antimicrobial Susceptibility Testing
CLSI	Clinical and Laboratory Standards Institute
EFSA	European Food Safety Authority
ESBL	Extended-spectrum β-lactamase
EU	European Union
EUCAST	European Committee on Antimicrobial Susceptibility Testing
FDA	Food and Drug Administration
FTIR	Fourier Transform Infrared
FT-RIFS	Fourier Transform Reflectometric Interference Spectroscopy
GMO	Genetically Modified Organism
HAD	Helicase-Dependent Amplification
HT-qPCR	High Throughput Quantitative PCR
IR	Infrared
IVD	in vitro Diagnostic
KPC	*Klebsiella pneumoniae* Carbapenemase
LAMP	Loop-Mediated Isothermal Amplification
LoC	Lab-on-a-Chip
LoD	limit of detection
MALDI-TOF MS	Matrix-Assisted Laser Desorption/Ionization Time-Of-Flight Mass Spectrometry
MCA	Morphokinetic Cellular Analysis
MDR	Multidrug-Resistant
MIC	Minimum Inhibitory Concentration
MLST	multi-locus sequence typing
MRSA	methicillin resistant *S. aureus*
MSSA	methicillin susceptible *S. aureus*
MZO	Magnesium Zinc Oxide
NAAT	Nucleic Acid Amplification Technology
NASBA	Nucleic Acid Sequence-Based Amplification
NGS	Next-Generation Sequencing
PCR	Polymerase Chain Reaction
PMF	Peptide Mass Fingerprint
POC	Point-of-Care
QCM	Quartz-Crystal Microbalance
RAA	Residual Antimicrobial Activity
RCA	Rolling Circle Amplification
RDT	Rapid Diagnostic Test
RPA	Recombinase Polymerase Amplification
SDA	Strand Displacement Amplification
SERS	Surface Enhanced Raman Spectroscopy
TAT	Turnaround Time
TMA	Transcription Mediated Amplification
UTI	Urinary Tract Infection
WGS	Whole Genomic Sequencing
WHO	World Health Organization
WMS	Whole Metagenome Sequencing

## References

[1] McAdams D., Wollein Waldetoft K., Tedijanto C., Lipsitch M., Brown S.P. (2019). Resistance diagnostics as a public health tool to combat antibiotic resistance: A model-based evaluation. PLoS Biol..

[2] Collignon P.C., Conly J.M., Andremont A., McEwen S.A., Aidara-Kane A., Agerso Y., Andremont A., Collignon P., Conly J., for the World Health Organization Advisory Group, Bogotá Meeting on Integrated Surveillance of Antimicrobial Resistance (WHO-AGISAR) (2016). World Health Organization Ranking of Antimicrobials According to Their Importance in Human Medicine: A Critical Step for Developing Risk Management Strategies to Control Antimicrobial Resistance From Food Animal Production. Clin. Infect. Dis..

[3] Leonard H., Colodner R., Halachmi S., Segal E. (2018). Recent Advances in the Race to Design a Rapid Diagnostic Test for Antimicrobial Resistance. Acs Sens..

[4] Ferri M., Ranucci E., Romagnoli P., Giaccone V. (2017). Antimicrobial resistance: A global emerging threat to public health systems. Crit. Rev. Food Sci. Nutr..

[5] Hashempour-Baltork F., Hosseini H., Shojaee-Aliabadi S., Torbati M., Alizadeh A.M., Alizadeh M. (2019). Drug Resistance and the Prevention Strategies in Food Borne Bacteria: An Update Review. Adv. Pharm. Bull..

[6] Friedman N.D., Temkin E., Carmeli Y. (2016). The negative impact of antibiotic resistance. Clin. Microbiol. Infect..

[7] Roope L.S.J., Smith R.D., Pouwels K.B., Buchanan J., Abel L., Eibich P., Butler C.C., Tan P.S., Walker A.S., Robotham J.V. (2019). The challenge of antimicrobial resistance: What economics can contribute. Science.

[8] O’Neill J. (2016). Tackling Drug-Resistant Infections Globally: Final Report and Recommendations.

[9] Bell G., MacLean C. (2018). The Search for ’Evolution-Proof’ Antibiotics. Trends Microbiol..

[10] Anderson M., Clift C., Schulze K., Sagan A., Nahrgang S., Ouakrim D.A., Mossialos E. (2019). Averting the AMR Crisis: What Are the Avenues for Policy Action for Countries in Europe?.

[11] Raoult D., Leone M., Roussel Y., Rolain J.-M. (2019). Attributable deaths caused by infections with antibiotic-resistant bacteria in France. Lancet Infect. Dis..

[12] Dadgostar P. (2019). Antimicrobial Resistance: Implications and Costs. Infect. Drug Resist..

[13] Ben Y., Fu C., Hu M., Liu L., Wong M.H., Zheng C. (2019). Human health risk assessment of antibiotic resistance associated with antibiotic residues in the environment: A review. Environ. Res..

[14] Rafiqi F. (2020). Antimicrobial Resistance Benchmark 2020.

[15] Cansizoglu M.F., Tamer Y.T., Farid M., Koh A.Y., Toprak E. (2019). Rapid ultrasensitive detection platform for antimicrobial susceptibility testing. PLoS Biol..

[16] Dubourg G., Raoult D. (2016). Emerging methodologies for pathogen identification in positive blood culture testing. Expert Rev. Mol. Diagn..

[17] Giordano C., Piccoli E., Brucculeri V., Barnini S. (2018). A Prospective Evaluation of Two Rapid Phenotypical Antimicrobial Susceptibility Technologies for the Diagnostic Stewardship of Sepsis. Biomed Res. Int..

[18] Cals J.W.L., Ament A.J.H.A., Hood K., Butler C.C., Hopstaken R.M., Wassink G.F., Dinant G.-J. (2011). C-reactive protein point of care testing and physician communication skills training for lower respiratory tract infections in general practice: Economic evaluation of a cluster randomized trial. J. Eval. Clin. Pract..

[19] Holmes E.A.F., Harris S.D., Hughes A., Craine N., Hughes D.A. (2018). Cost-Effectiveness Analysis of the Use of Point-of-Care C-Reactive Protein Testing to Reduce Antibiotic Prescribing in Primary Care. Antibiotics.

[20] Hunter R. (2015). Cost-effectiveness of point-of-care C-reactive protein tests for respiratory tract infection in primary care in England. Adv. Ther..

[21] Richter S.S., Karichu J., Otiso J., Van Heule H., Keller G., Cober E., Rojas L.J., Hujer A.M., Hujer K.M., Marshall S. (2018). Evaluation of Sensititre Broth Microdilution Plate for determining the susceptibility of carbapenem-resistant Klebsiella pneumoniae to polymyxins. Diagn. Microbiol. Infect. Dis..

[22] Álvarez-Molina A., de Toro M., Alexa E.A., Álvarez-Ordóñez A. (2020). Applying Genomics to Track Antimicrobial Resistance in the Food Chain. Ref. Modul. Food Sci..

[23] Andrews J.M. (2001). Determination of minimum inhibitory concentrations. J. Antimicrob. Chemother..

[24] Kahlmeter G., Brown D., Finch R.G., Greenwood D., Norrby S.R., Whitley R.J. (2010). CHAPTER 9—Laboratory control of antimicrobial therapy. Antibiotic and Chemotherapy.

[25] Tenover F.C., Schaechter M. (2009). Antibiotic Susceptibility Testing. Encyclopedia of Microbiology.

[26] McGill K., Kelly L., Madden R.H., Moran L., Carroll C., O’Leary A., Moore J.E., McNamara E., O’Mahony M., Fanning S. (2009). Comparison of disc diffusion and epsilometer (E-test) testing techniques to determine antimicrobial susceptibiliy of *Campylobacter* isolates of food and human clinical origin. J. Microbiol. Methods.

[27] Vasala A., Hytönen V.P., Laitinen O.H. (2020). Modern Tools for Rapid Diagnostics of Antimicrobial Resistance. Front. Cell. Infect. Microbiol..

[28] Fluit A.C., Visser M.R., Schmitz F.-J. (2001). Molecular Detection of Antimicrobial Resistance. Clin. Microbiol. Rev..

[29] Sundsfjord A., Simonsen G.S., Haldorsen B.C., Haaheim H., Hjelmevoll S.-O., Littauer P.I.A., Dahl K.H. (2004). Genetic methods for detection of antimicrobial resistance. APMIS.

[30] Waseem H., Jameel S., Ali J., Saleem Ur Rehman H., Tauseef I., Farooq U., Jamal A., Ali M.I. (2019). Contributions and Challenges of High Throughput qPCR for Determining Antimicrobial Resistance in the Environment: A Critical Review. Molecules.

[31] Chisholm S.A., Owen R.J., Teare E.L., Saverymuttu S. (2001). PCR-Based Diagnosis of *Helicobacter pylori* Infection and Real-Time Determination of Clarithromycin Resistance Directly from Human Gastric Biopsy Samples. J. Clin. Microbiol..

[32] Seedy F.R.E., Samy A.A., Salam H.S.H., Khairy E.A., Koraney A.A. (2017). Polymerase chain reaction detection of genes responsible for multiple antibiotic resistance *Staphylococcus aureus* isolated from food of animal origin in Egypt. Vet. World.

[33] Mackay I.M. (2004). Real-time PCR in the microbiology laboratory. Clin. Microbiol. Infect..

[34] Böckelmann U., Dörries H.-H., Ayuso-Gabella M.N., de Salgot Marçay M., Tandoi V., Levantesi C., Masciopinto C., Van Houtte E., Szewzyk U., Wintgens T. (2009). Quantitative PCR Monitoring of Antibiotic Resistance Genes and Bacterial Pathogens in Three European Artificial Groundwater Recharge Systems. Appl. Environ. Microbiol..

[35] Whale A.S., Bushell C.A., Grant P.R., Cowen S., Gutierrez-Aguirre I., Sullivan D.M., Žel J., Milavec M., Foy C.A., Nastouli E. (2016). Detection of Rare Drug Resistance Mutations by Digital PCR in a Human Influenza A Virus Model System and Clinical Samples. J. Clin. Microbiol..

[36] Bogožalec Košir A., Cvelbar T., Kammel M., Grunert H.-P., Zeichhardt H., Milavec M. (2020). Digital PCR method for detection and quantification of specific antimicrobial drug-resistance mutations in human *cytomegalovirus*. J. Virol. Methods.

[37] Jousset A.B., Bernabeu S., Bonnin R.A., Creton E., Cotellon G., Sauvadet A., Naas T., Dortet L. (2019). Development and validation of a multiplex polymerase chain reaction assay for detection of the five families of plasmid-encoded colistin resistance. Int. J. Antimicrob. Agents.

[38] Waseem H., Saleem ur Rehman H., Ali J., Iqbal M.J., Ali M.I., Hashmi M.Z. (2020). Chapter 14—Global trends in ARGs measured by HT-qPCR platforms. Antibiotics and Antimicrobial Resistance Genes in the Environment.

[39] Wang F.-H., Qiao M., Su J.-Q., Chen Z., Zhou X., Zhu Y.-G. (2014). High Throughput Profiling of Antibiotic Resistance Genes in Urban Park Soils with Reclaimed Water Irrigation. Environ. Sci. Technol..

[40] Phuklia W., Panyanivong P., Sengdetka D., Sonthayanon P., Newton P.N., Paris D.H., Day N.P.J., Dittrich S. (2018). Novel high-throughput screening method using quantitative PCR to determine the antimicrobial susceptibility of *Orientia tsutsugamushi* clinical isolates. J. Antimicrob. Chemother..

[41] Xu L., Chen H., Canales M., Ciric L. (2019). Use of synthesized double-stranded gene fragments as qPCR standards for the quantification of antibiotic resistance genes. J. Microbiol. Methods.

[42] Donà V., Kasraian S., Lupo A., Guilarte Y.N., Hauser C., Furrer H., Unemo M., Low N., Endimiani A. (2016). Multiplex Real-Time PCR Assay with High-Resolution Melting Analysis for Characterization of Antimicrobial Resistance in *Neisseria gonorrhoeae*. J. Clin. Microbiol..

[43] Wang H., Hecht S., Kline D., Leber A.L. (2019). *Staphylococcus aureus* and methicillin resistance detection directly from pediatric samples using PCR assays with differential cycle threshold values for corroboration of methicillin resistance. J. Microbiol. Methods.

[44] Masny A., Płucienniczak A. (2003). Ligation mediated PCR performed at low denaturation temperatures—PCR melting profiles. Nucleic Acids Res..

[45] Woksepp H., Jernberg C., Tärnberg M., Ryberg A., Brolund A., Nordvall M., Olsson-Liljequist B., Wisell K.T., Monstein H.-J., Nilsson L.E. (2011). High-resolution melting-curve analysis of ligation-mediated real-time PCR for rapid evaluation of an epidemiological outbreak of extended-spectrum-beta-lactamase-producing Escherichia coli. J. Clin. Microbiol..

[46] Nijhuis R., van Zwet A., Stuart J.C., Weijers T., Savelkoul P. (2012). Rapid molecular detection of extended-spectrum β-lactamase gene variants with a novel ligation-mediated real-time PCR. J. Med. Microbiol..

[47] Woksepp H., Ryberg A., Billström H., Hällgren A., Nilsson L.E., Marklund B.-I., Olsson-Liljequist B., Schön T. (2014). Evaluation of high-resolution melting curve analysis of ligation-mediated real-time PCR, a rapid method for epidemiological typing of ESKAPE (Enterococcus faecium, Staphylococcus aureus, Klebsiella pneumoniae, Acinetobacter baumannii, Pseudomonas aeruginosa, and Enterobacter species) pathogens. J. Clin. Microbiol..

[48] Gill P., Ghaemi A. (2008). Nucleic acid isothermal amplification technologies: A review. Nucleosidesnucleotides Nucleic Acids.

[49] Lee S.H., Park S.-M., Kim B.N., Kwon O.S., Rho W.-Y., Jun B.-H. (2019). Emerging ultrafast nucleic acid amplification technologies for next-generation molecular diagnostics. Biosens. Bioelectron..

[50] Zou Y., Mason M.-G., Botella J.-R. (2020). Evaluation and improvement of isothermal amplification methods for point-of-need plant disease diagnostics. PLoS ONE.

[51] Zanoli L.M., Spoto G. (2012). Isothermal amplification methods for the detection of nucleic acids in microfluidic devices. Biosensors.

[52] Karami A., Gill P., Motamedi M., Saghafinia M. (2011). A review of the current isothermal amplification techniques: Applications, advantages and disadvantages. J. Glob. Infect. Dis..

[53] Kaprou G.D., Papadakis G., Papageorgiou D.P., Kokkoris G., Papadopoulos V., Kefala I., Gizeli E., Tserepi A. (2016). Miniaturized devices for isothermal DNA amplification addressing DNA diagnostics. Microsyst. Technol..

[54] Srimongkol G., Ditmangklo B., Choopara I., Thaniyavarn J., Dean D., Kokpol S., Vilaivan T., Somboonna N. (2020). Rapid colorimetric loop-mediated isothermal amplification for hypersensitive point-of-care *Staphylococcus aureus* enterotoxin A gene detection in milk and pork products. Sci. Rep..

[55] Dhama K., Karthik K., Chakraborty S., Tiwari R., Kapoor S., Kumar A., Thomas P. (2016). Loop-mediated isothermal amplification of DNA (LAMP): A new diagnostic tool lights the world of diagnosis of animal and human pathogens: A review. Pak. J. Biol. Sci..

[56] Morin S., Bazarova N., Jacon P., Vella S. (2017). The Manufacturers’ Perspective on World Health Organization Prequalification of In Vitro Diagnostics. Clin. Infect. Dis..

[57] Cantera J.L., White H., Diaz M.H., Beall S.G., Winchell J.M., Lillis L., Kalnoky M., Gallarda J., Boyle D.S. (2019). Assessment of eight nucleic acid amplification technologies for potential use to detect infectious agents in low-resource settings. PLoS ONE.

[58] Call D.R., Bakko M.K., Krug M.J., Roberts M.C. (2003). Identifying antimicrobial resistance genes with DNA microarrays. Antimicrob. Agents Chemother..

[59] Anjum M.F., Zankari E., Hasman H. (2017). Molecular Methods for Detection of Antimicrobial Resistance. Microbiol. Spectr..

[60] Strauss C., Endimiani A., Perreten V. (2015). A novel universal DNA labeling and amplification system for rapid microarray-based detection of 117 antibiotic resistance genes in Gram-positive bacteria. J. Microbiol. Methods.

[61] Zhu L.-X., Zhang Z.-W., Wang C., Yang H.-W., Jiang D., Zhang Q., Mitchelson K., Cheng J. (2007). Use of a DNA Microarray for Simultaneous Detection of Antibiotic Resistance Genes among *Staphylococcal* Clinical Isolates. J. Clin. Microbiol..

[62] Havlicek J., Dachsel B., Slickers P., Andres S., Beckert P., Feuerriegel S., Niemann S., Merker M., Labugger I. (2019). Rapid microarray-based assay for detection of pyrazinamide resistant *Mycobacterium tuberculosis*. Diagn. Microbiol. Infect. Dis..

[63] Sanger F., Nicklen S., Coulson A.R. (1977). DNA sequencing with chain-terminating inhibitors. Proc. Natl. Acad. Sci. USA.

[64] Maxam A.M., Gilbert W. (1977). A new method for sequencing DNA. Proc. Natl. Acad. Sci. USA.

[65] Fleischmann R.D., Adams M.D., White O., Clayton R.A., Kirkness E.F., Kerlavage A.R., Bult C.J., Tomb J.F., Dougherty B.A., Merrick J.M. (1995). Whole-genome random sequencing and assembly of *Haemophilus* influenzae Rd. Science.

[66] Margulies M., Egholm M., Altman W.E., Attiya S., Bader J.S., Bemben L.A., Berka J., Braverman M.S., Chen Y.-J., Chen Z. (2005). Genome sequencing in microfabricated high-density picolitre reactors. Nature.

[67] Teng J.L.L., Yeung M.L., Chan E., Jia L., Lin C.H., Huang Y., Tse H., Wong S.S.Y., Sham P.C., Lau S.K.P. (2017). PacBio But Not Illumina Technology Can Achieve Fast, Accurate and Complete Closure of the High GC, Complex *Burkholderia pseudomallei* Two-Chromosome Genome. Front. Microbiol..

[68] Levene M.J., Korlach J., Turner S.W., Foquet M., Craighead H.G., Webb W.W. (2003). Zero-Mode Waveguides for Single-Molecule Analysis at High Concentrations. Science.

[69] Clarke J., Wu H.-C., Jayasinghe L., Patel A., Reid S., Bayley H. (2009). Continuous base identification for single-molecule nanopore DNA sequencing. Nat. Nanotechnol..

[70] Ruppé E., Bengtsson-Palme J., Charretier Y., Schrenzel J. (2019). How next-generation sequencing can address the antimicrobial resistance challenge. AMR Control.

[71] Malmberg C., Yuen P., Spaak J., Cars O., Tängdén T., Lagerbäck P. (2016). A Novel Microfluidic Assay for Rapid Phenotypic Antibiotic Susceptibility Testing of Bacteria Detected in Clinical Blood Cultures. PLoS ONE.

[72] Köser C.U., Ellington M.J., Peacock S.J. (2014). Whole-genome sequencing to control antimicrobial resistance. Trends Genet..

[73] Oniciuc E.A., Likotrafiti E., Alvarez-Molina A., Prieto M., Santos J.A., Alvarez-Ordóñez A. (2018). The Present and Future of Whole Genome Sequencing (WGS) and Whole Metagenome Sequencing (WMS) for Surveillance of Antimicrobial Resistant Microorganisms and Antimicrobial Resistance Genes across the Food Chain. Genes.

[74] Hendriksen R.S., Bortolaia V., Tate H., Tyson G.H., Aarestrup F.M., McDermott P.F. (2019). Using Genomics to Track Global Antimicrobial Resistance. Front. Public Health.

[75] Boolchandani M., D’Souza A.W., Dantas G. (2019). Sequencing-based methods and resources to study antimicrobial resistance. Nat. Rev. Genet..

[76] Zankari E., Allesøe R., Joensen K.G., Cavaco L.M., Lund O., Aarestrup F.M. (2017). PointFinder: A novel web tool for WGS-based detection of antimicrobial resistance associated with chromosomal point mutations in bacterial pathogens. J Antimicrob Chemother.

[77] Mason A., Foster D., Bradley P., Golubchik T., Doumith M., Gordon N.C., Pichon B., Iqbal Z., Staves P., Crook D. (2018). Accuracy of Different Bioinformatics Methods in Detecting Antibiotic Resistance and Virulence Factors from *Staphylococcus aureus* Whole-Genome Sequences. J. Clin. Microbiol..

[78] Lauener F.N., Imkamp F., Lehours P.A.-O., Buissonnière A., Benejat L., Zbinden R., Keller P.A.-O., Wagner K. (2019). Genetic Determinants and Prediction of Antibiotic Resistance Phenotypes in *Helicobacter pylori*. J. Clin. Med..

[79] Su M., Satola S.W., Read T.D. (2019). Genome-Based Prediction of Bacterial Antibiotic Resistance. J. Clin. Microbiol..

[80] Nguyen M., Long S.W., McDermott P.F., Olsen R.J., Olson R., Stevens R.L., Tyson G.H., Zhao S., Davis J.J. (2019). Using Machine Learning to Predict Antimicrobial MICs and Associated Genomic Features for Nontyphoidal *Salmonella*. J. Clin. Microbiol..

[81] Hyun J.C., Kavvas E.S., Monk J.M., Palsson B.O. (2020). Machine learning with random subspace ensembles identifies antimicrobial resistance determinants from pan-genomes of three pathogens. PLoS Comput. Biol..

[82] González-Escalona N.A.O., Allard M.A., Brown E.W., Sharma S., Hoffmann M. (2019). Nanopore sequencing for fast determination of plasmids, phages, virulence markers, and antimicrobial resistance genes in Shiga toxin-producing *Escherichia coli*. PLoS ONE.

[83] Amoako K.K., Thomas M.C., Kong F., Janzen T.W., Hahn K.R., Shields M.J., Goji N. (2012). Rapid detection and antimicrobial resistance gene profiling of *Yersinia pestis* using pyrosequencing technology. J. Microbiol. Methods.

[84] Ajbani K., Lin S.Y., Rodrigues C., Nguyen D., Arroyo F., Kaping J., Jackson L., Garfein R.S., Catanzaro D., Eisenach K. (2015). Evaluation of pyrosequencing for detecting extensively drug-resistant *Mycobacterium tuberculosis* among clinical isolates from four high-burden countries. Antimicrob. Agents Chemother..

[85] Govindappa M., Farheen H., Chandrappa C.P., Rai R.V., Raghavendra V.B. (2016). Mycosynthesis of silver nanoparticles using extract of endophytic fungi, Penicillium species of *Glycosmis mauritiana*, and its antioxidant, antimicrobial, anti-inflammatory and tyrokinase inhibitory activity. Adv. Nat. Sci. Nanosci. Nanotechnol..

[86] McDermott P.F., Tyson G.H., Kabera C., Chen Y., Li C., Folster J.P., Ayers S.L., Lam C., Tate H.P., Zhao S. (2016). Whole-genome sequencing for detecting antimicrobial resistance in nontyphoidal *Salmonella*. Antimicrob. Agents Chemother..

[87] Vélez J.R., Cameron M., Rodríguez-Lecompte J.C., Xia F., Heider L.C., Saab M., McClure J.T., Sánchez J. (2017). Whole-Genome Sequence Analysis of Antimicrobial Resistance Genes in *Streptococcus uberis* and *Streptococcus dysgalactiae* Isolates from Canadian Dairy Herds. Front. Vet. Sci..

[88] Zhao S., Tyson G.H., Chen Y., Li C., Mukherjee S., Young S., Lam C., Folster J.P., Whichard J.M., McDermott P.F. (2016). Whole-Genome Sequencing Analysis Accurately Predicts Antimicrobial Resistance Phenotypes in *Campylobacter* Spp.. Appl. Environ. Microbiol..

[89] Alghoribi M.F., Balkhy H.H., Woodford N., Ellington M.J. (2018). The role of whole genome sequencing in monitoring antimicrobial resistance: A biosafety and public health priority in the Arabian Peninsula. J. Infect. Public Health.

[90] Argimón S., Masim M.A.L., Gayeta J.M., Lagrada M.L., Macaranas P.K.V., Cohen V., Limas M.T., Espiritu H.O., Palarca J.C., Chilam J. (2020). Integrating whole-genome sequencing within the National Antimicrobial Resistance Surveillance Program in the Philippines. Nat. Commun..

[91] Fasciana T., Gentile B., Aquilina M., Ciammaruconi A., Mascarella C., Anselmo A., Fortunato A., Fillo S., Petralito G., Lista F. (2019). Co-existence of virulence factors and antibiotic resistance in new Klebsiella pneumoniae clones emerging in south of Italy. BMC Infect. Dis..

[92] Berbers B., Saltykova A., Garcia-Graells C., Philipp P., Arella F., Marchal K., Winand R., Vanneste K., Roosens N.H.C., De Keersmaecker S.C.J. (2020). Combining short and long read sequencing to characterize antimicrobial resistance genes on plasmids applied to an unauthorized genetically modified *Bacillus*. Sci. Rep..

[93] Quick J., Loman N.J., Duraffour S., Simpson J.T., Severi E., Cowley L., Bore J.A., Koundouno R., Dudas G., Mikhail A. (2016). Real-time, portable genome sequencing for Ebola surveillance. Nature.

[94] Giordano F., Aigrain L., Quail M.A., Coupland P., Bonfield J.K., Davies R.M., Tischler G., Jackson D.K., Keane T.M., Li J. (2017). De novo yeast genome assemblies from MinION, PacBio and MiSeq platforms. Sci. Rep..

[95] Loman N.J., Quick J., Simpson J.T. (2015). A complete bacterial genome assembled de novo using only nanopore sequencing data. Nat. Methods.

[96] Greninger A.L., Naccache S.N., Federman S., Yu G., Mbala P., Bres V., Stryke D., Bouquet J., Somasekar S., Linnen J.M. (2015). Rapid metagenomic identification of viral pathogens in clinical samples by real-time nanopore sequencing analysis. Genome Med..

[97] Brown B.L., Watson M., Minot S.S., Rivera M.C., Franklin R.B. (2017). MinION™ nanopore sequencing of environmental metagenomes: A synthetic approach. GigaScience.

[98] Judge K., Harris S.R., Reuter S., Parkhill J., Peacock S.J. (2015). Early insights into the potential of the Oxford Nanopore MinION for the detection of antimicrobial resistance genes. J. Antimicrob. Chemother..

[99] Ashton P.M., Nair S., Dallman T., Rubino S., Rabsch W., Mwaigwisya S., Wain J., O’Grady J. (2015). MinION nanopore sequencing identifies the position and structure of a bacterial antibiotic resistance island. Nat. Biotechnol..

[100] Greig D.R., Dallman T.J., Hopkins K.L., Jenkins C. (2018). MinION nanopore sequencing identifies the position and structure of bacterial antibiotic resistance determinants in a multidrug-resistant strain of enteroaggregative *Escherichia coli*. Microb. Genom..

[101] Schmidt K., Mwaigwisya S., Crossman L.C., Doumith M., Munroe D., Pires C., Khan A.M., Woodford N., Saunders N.J., Wain J. (2016). Identification of bacterial pathogens and antimicrobial resistance directly from clinical urines by nanopore-based metagenomic sequencing. J. Antimicrob. Chemother..

[102] Kamathewatta K.I., Bushell R.N., Young N.D., Stevenson M.A., Billman-Jacobe H., Browning G.F., Marenda M.S. (2019). Exploration of antibiotic resistance risks in a veterinary teaching hospital with Oxford Nanopore long read sequencing. PLoS ONE.

[103] Taxt A.M., Avershina E., Frye S.A., Naseer U., Ahmad R. (2020). Rapid identification of pathogens, antibiotic resistance genes and plasmids in blood cultures by nanopore sequencing. Sci. Rep..

[104] Tan S., Dvorak C.M.T., Estrada A.A., Gebhart C., Marthaler D.G., Murtaugh M.P. (2020). MinION sequencing of *Streptococcus suis* allows for functional characterization of bacteria by multilocus sequence typing and antimicrobial resistance profiling. J. Microbiol. Methods.

[105] Lim A., Naidenov B., Bates H., Willyerd K., Snider T., Couger M.B., Chen C., Ramachandran A. (2019). Nanopore ultra-long read sequencing technology for antimicrobial resistance detection in *Mannheimia haemolytica*. J. Microbiol. Methods.

[106] Angers A., Petrillo M., Patak D.A., Querci M., Guy V.D.E. (2017). The Role and Implementation of Next-Generation Sequencing Technologies in the Coordinated Action Plan against Antimicrobial Resistance.

[107] Gajdács M., Spengler G., Urbán E. (2017). Identification and Antimicrobial Susceptibility Testing of Anaerobic Bacteria: Rubik’s Cube of Clinical Microbiology?. Antibiotics.

[108] Justesen U.S., Acar Z., Sydenham T.V., Johansson Å. (2018). Antimicrobial susceptibility testing of Bacteroides fragilis using the MALDI Biotyper antibiotic susceptibility test rapid assay (MBT-ASTRA). Anaerobe.

[109] Zürcher S., Mooser C., Lüthi A.U., Mühlemann K., Barbani M.T., Mohacsi P., Garzoni C., Gorgievski-Hrisoho M., Schaller A., Flatz L. (2012). Sensitive and rapid detection of ganciclovir resistance by PCR based MALDI-TOF analysis. J. Clin. Virol..

[110] Liu N., Wang L., Cai G., Zhang D., Lin J. (2019). Establishment of a simultaneous detection method for ten duck viruses using MALDI-TOF mass spectrometry. J. Virol. Methods.

[111] Paul S., Singh P., As S., Rudramurthy S.M., Chakrabarti A., Ghosh A.K. (2017). Rapid detection of fluconazole resistance in *Candida tropicalis* by MALDI-TOF MS. Med. Mycol..

[112] Angeletti S. (2017). Matrix assisted laser desorption time of flight mass spectrometry (MALDI-TOF MS) in clinical microbiology. J. Microbiol. Methods.

[113] Welker M., van Belkum A. (2019). One System for All: Is Mass Spectrometry a Future Alternative for Conventional Antibiotic Susceptibility Testing?. Front. Microbiol..

[114] Vrioni G., Tsiamis C., Oikonomidis G., Theodoridou K., Kapsimali V., Tsakris A. (2018). MALDI-TOF mass spectrometry technology for detecting biomarkers of antimicrobial resistance: Current achievements and future perspectives. Ann. Transl. Med..

[115] Singhal N., Kumar M., Kanaujia P.K., Virdi J.S. (2015). MALDI-TOF mass spectrometry: An emerging technology for microbial identification and diagnosis. Front. Microbiol..

[116] Hoyos-Mallecot Y., Riazzo C., Miranda-Casas C., Rojo-Martín M.D., Gutiérrez-Fernández J., Navarro-Marí J.M. (2014). Rapid detection and identification of strains carrying carbapenemases directly from positive blood cultures using MALDI-TOF MS. J. Microbiol. Methods.

[117] Rodríguez-Sánchez B., Cercenado E., Coste A.T., Greub G. (2019). Review of the impact of MALDI-TOF MS in public health and hospital hygiene, 2018. Eurosurveill.

[118] Wang K., Li S., Petersen M., Wang S., Lu X. (2018). Detection and Characterization of Antibiotic-Resistant Bacteria Using Surface-Enhanced Raman Spectroscopy. Nanomaterials.

[119] Byun J.H., Yu A.R., Kim M.S., Lee K. (2018). Performance of Microflex LT Biotyper and VITEK MS for Routine Identification of Yeasts. Ann. Lab Med..

[120] Martiny D., Busson L., Wybo I., El Haj R.A., Dediste A., Vandenberg O. (2012). Comparison of the Microflex LT and Vitek MS systems for routine identification of bacteria by matrix-assisted laser desorption ionization-time of flight mass spectrometry. J. Clin. Microbiol..

[121] Marko D.C., Saffert R.T., Cunningham S.A., Hyman J., Walsh J., Arbefeville S., Howard W., Pruessner J., Safwat N., Cockerill F.R. (2012). Evaluation of the Bruker Biotyper and Vitek MS matrix-assisted laser desorption ionization-time of flight mass spectrometry systems for identification of nonfermenting gram-negative bacilli isolated from cultures from cystic fibrosis patients. J. Clin. Microbiol..

[122] Dortet L., Bonnin R.A., Pennisi I., Gauthier L., Jousset A.B., Dabos L., Furniss R.C.D., Mavridou D.A.I., Bogaerts P., Glupczynski Y. (2018). Rapid detection and discrimination of chromosome- and MCR-plasmid-mediated resistance to polymyxins by MALDI-TOF MS in *Escherichia coli*: The MALDIxin test. J Antimicrob Chemother.

[123] Furniss R.A.-O., Dortet L.A.-O., Bolland W., Drews O., Sparbier K., Bonnin R.A., Filloux A.A.-O., Kostrzewa M., Mavridou D.A.-O., Larrouy-Maumus G. (2019). Detection of Colistin Resistance in *Escherichia coli* by Use of the MALDI Biotyper Sirius Mass Spectrometry System. J. Clin. Microbiol..

[124] Kawamoto Y., Kosai K., Yamakawa H., Kaku N., Uno N., Morinaga Y., Hasegawa H., Yanagihara K. (2019). Detection of extended-spectrum β-lactamase (ESBL)-producing *Enterobacteriaceae* using the MALDI Biotyper Selective Testing of Antibiotic Resistance–β-Lactamase (MBT STAR-BL) assay. J. Microbiol. Methods.

[125] Nisa S., Bercker C., Midwinter A.C., Bruce I., Graham C.F., Venter P., Bell A., French N.P., Benschop J., Bailey K.M. (2019). Combining MALDI-TOF and genomics in the study of methicillin resistant and multidrug resistant *Staphylococcus pseudintermedius* in New Zealand. Sci. Rep..

[126] Tang W., Ranganathan N., Shahrezaei V., Larrouy-Maumus G. (2019). MALDI-TOF mass spectrometry on intact bacteria combined with a refined analysis framework allows accurate classification of MSSA and MRSA. PLoS ONE.

[127] Vatanshenassan M., Boekhout T., Meis J.F., Berman J., Chowdhary A., Ben-Ami R., Sparbier K., Kostrzewa M. (2019). *Candida auris* Identification and Rapid Antifungal Susceptibility Testing Against Echinocandins by MALDI-TOF MS. Front. Cell. Infect. Microbiol..

[128] Cordovana M., Pranada A.B., Ambretti S., Kostrzewa M. (2019). MALDI-TOF bacterial subtyping to detect antibiotic resistance. Clin. Mass Spectrom..

[129] Ota Y., Furuhashi K., Nagao Y., Nanba T., Yamanaka K., Ishikawa J., Nagura O., Iwaizumi M., Hamada E., Maekawa M. (2019). Detection of extended-spectrum β-lactamases producing *Enterobacteriaceae* using a matrix-assisted laser desorption/ionization time-of-flight mass spectrometry based MBT STAR-BL software module with β-lactamase inhibition assay depends on the bacterial strains. J. Microbiol. Methods.

[130] Neonakis I.K., Spandidos D.A. (2019). Detection of carbapenemase producers by matrix-assisted laser desorption-ionization time-of-flight mass spectrometry (MALDI-TOF MS). Eur. J. Clin. Microbiol. Infect Dis..

[131] Li J., Hu W., Li M., Deng S., Huang Q., Lu W. (2019). Evaluation of matrix-assisted laser desorption/ionization time-of-flight mass spectrometry for identifying VIM- and SPM-type metallo-β-lactamase-producing *Pseudomonas aeruginosa* clinical isolates. Infect. Drug Resist..

[132] Parlapani F.F., Kyritsi M., Sakka M., Chatzinikolaou K., Donos S., Boziaris I.S., Hadjichristodoulou C., Athanassiou C.G. (2020). Matrix-assisted laser desorption ionization–time of flight mass spectrometry reveals *Enterococcus* and *Enterobacter spp*. in major insect species involved in food security with resistance to common antibiotics. J. Pest Sci..

[133] Rocco V.G., Intra J., Sarto C., Tiberti N., Savarino C., Brambilla M., Brambilla P. (2019). Rapid Identification of Carbapenemase-producing *Klebsiella pneumoniae* strains by Matrix-Assisted Laser Desorption/Ionization-Time of Flight using Vitek(®) Mass Spectrometry System. Eurasian J. Med..

[134] Dortet L.A.-O., Potron A., Bonnin R.A.-O., Plesiat P., Naas T.A.-O., Filloux A.A.-O., Larrouy-Maumus G. (2018). Rapid detection of colistin resistance in *Acinetobacter baumannii* using MALDI-TOF-based lipidomics on intact bacteria. Sci. Rep..

[135] Axelsson C., Rehnstam-Holm A.-S., Nilson B. (2020). Rapid detection of antibiotic resistance in positive blood cultures by MALDI-TOF MS and an automated and optimized MBT-ASTRA protocol for *Escherichia coli* and *Klebsiella pneumoniae*. Infect. Dis..

[136] Horseman T.S., Lustik M.B., Fong K.S.K. (2020). Rapid qualitative antibiotic resistance characterization using VITEK MS. Diagn. Microbiol. Infect. Dis..

[137] Oviaño M., Gato E., Bou G. (2020). Rapid Detection of KPC-Producing *Enterobacterales* Susceptible to Imipenem/Relebactam by Using the MALDI-TOF MS MBT STAR-Carba IVD Assay. Front. Microbiol..

[138] Nix I.D., Idelevich E.A., Storck L.M., Sparbier K., Drews O., Kostrzewa M., Becker K. (2020). Detection of Methicillin Resistance in *Staphylococcus aureus* From Agar Cultures and Directly From Positive Blood Cultures Using MALDI-TOF Mass Spectrometry-Based Direct-on-Target Microdroplet Growth Assay. Front. Microbiol..

[139] Vogt S., Löffler K., Dinkelacker A.G., Bader B., Autenrieth I.B., Peter S., Liese J. (2019). Fourier-Transform Infrared (FTIR) Spectroscopy for Typing of Clinical *Enterobacter cloacae* Complex Isolates. Front. Microbiol..

[140] Salman A., Sharaha U., Rodriguez-Diaz E., Shufan E., Riesenberg K., Bigio I.J., Huleihel M. (2017). Detection of antibiotic resistant *Escherichia Coli* bacteria using infrared microscopy and advanced multivariate analysis. Analyst.

[141] Zwielly A., Gopas J., Brkic G., Mordechai S. (2009). Discrimination between drug-resistant and non-resistant human melanoma cell lines by FTIR spectroscopy. Analyst.

[142] Novais Â., Freitas A.R., Rodrigues C., Peixe L. (2019). Fourier transform infrared spectroscopy: Unlocking fundamentals and prospects for bacterial strain typing. Eur. J. Clin. Microbiol. Infect. Dis..

[143] Lechowicz L., Urbaniak M., Adamus-Białek W., Kaca W. (2013). The use of infrared spectroscopy and artificial neural networks for detection of uropathogenic *Escherichia coli* strains’ susceptibility to cephalothin. Acta Biochim. Pol..

[144] Sharaha U., Rodriguez-Diaz E., Riesenberg K., Bigio I.J., Huleihel M., Salman A. (2017). Using Infrared Spectroscopy and Multivariate Analysis to Detect Antibiotics’ Resistant *Escherichia coli* Bacteria. Anal. Chem..

[145] Kochan K., Nethercott C., Perez−Guaita D., Jiang J.-H., Peleg A.Y., Wood B.R., Heraud P. (2019). Detection of Antimicrobial Resistance-Related Changes in Biochemical Composition of *Staphylococcus aureus* by Means of Atomic Force Microscopy-Infrared Spectroscopy. Anal. Chem..

[146] Papadakis G., Pantazis A.K., Ntogka M., Parasyris K., Theodosi G.-I., Kaprou G., Gizeli E. (2019). 3D-printed Point-of-Care Platform for Genetic Testing of Infectious Diseases Directly in Human Samples Using Acoustic Sensors and a Smartphone. ACS Sens..

[147] Tsougeni K., Kastania A.S., Kaprou G.D., Eck M., Jobst G., Petrou P.S., Kakabakos S.E., Mastellos D., Gogolides E., Tserepi A. (2019). A modular integrated lab-on-a-chip platform for fast and highly efficient sample preparation for foodborne pathogen screening. Sens. Actuators B Chem..

[148] Fernández-Gavela A., Herranz S., Chocarro B., Falke F., Schreuder E., Leeuwis H., Heideman R.G., Lechuga L.M. (2019). Full integration of photonic nanoimmunosensors in portable platforms for on-line monitoring of ocean pollutants. Sens. Actuators B Chem..

[149] Yılmaz B., Yılmaz F., Barh D., Azevedo V. (2018). Chapter 8—Lab-on-a-Chip Technology and Its Applications. Omics Technologies and Bio-Engineering.

[150] Jung W., Han J., Choi J.-W., Ahn C.H. (2015). Point-of-care testing (POCT) diagnostic systems using microfluidic lab-on-a-chip technologies. Microelectron. Eng..

[151] Sackmann E.K., Fulton A.L., Beebe D.J. (2014). The present and future role of microfluidics in biomedical research. Nature.

[152] Kaprou G.D., Papadopoulos V., Papageorgiou D.P., Kefala I., Papadakis G., Gizeli E., Chatzandroulis S., Kokkoris G., Tserepi A. (2019). Ultrafast, low-power, PCB manufacturable, continuous-flow microdevice for DNA amplification. Anal. Bioanal. Chem..

[153] Zhang G., Zheng G., Zhang Y., Ma R., Kang X. (2018). Evaluation of a micro/nanofluidic chip platform for the high-throughput detection of bacteria and their antibiotic resistance genes in post-neurosurgical meningitis. Int. J. Infect. Dis..

[154] Giuffrida M.C., Spoto G. (2017). Integration of isothermal amplification methods in microfluidic devices: Recent advances. Biosens. Bioelectron..

[155] Matsumoto Y., Sakakihara S., Grushnikov A., Kikuchi K., Noji H., Yamaguchi A., Iino R., Yagi Y., Nishino K. (2016). A Microfluidic Channel Method for Rapid Drug-Susceptibility Testing of *Pseudomonas aeruginosa*. PLoS ONE.

[156] Dong T., Zhao X. (2015). Rapid Identification and Susceptibility Testing of Uropathogenic Microbes via Immunosorbent ATP-Bioluminescence Assay on a Microfluidic Simulator for Antibiotic Therapy. Anal. Chem..

[157] Choi J., Yoo J., Lee M., Kim E.G., Lee J.S., Lee S., Joo S., Song S.H., Kim E.C., Lee J.C. (2014). A rapid antimicrobial susceptibility test based on single-cell morphological analysis. Sci. Transl. Med..

[158] Pitruzzello G., Thorpe S., Johnson S., Evans A., Gadêlha H., Krauss T.F. (2019). Multiparameter antibiotic resistance detection based on hydrodynamic trapping of individual *E. coli*. Lab Chip.

[159] Galvan D.D., Yu Q. (2018). Surface-Enhanced Raman Scattering for Rapid Detection and Characterization of Antibiotic-Resistant Bacteria. Adv. Healthc. Mater..

[160] Chen X., Tang M., Liu Y., Huang J., Liu Z., Tian H., Zheng Y., de la Chapelle M.L., Zhang Y., Fu W. (2019). Surface-enhanced Raman scattering method for the identification of methicillin-resistant *Staphylococcus aureus* using positively charged silver nanoparticles. Microchim. Acta.

[161] Lu X., Samuelson D.R., Xu Y., Zhang H., Wang S., Rasco B.A., Xu J., Konkel M.E. (2013). Detecting and tracking nosocomial methicillin-resistant *Staphylococcus aureus* using a microfluidic SERS biosensor. Anal. Chem..

[162] Chang K.-W., Cheng H.-W., Shiue J., Wang J.-K., Wang Y.-L., Huang N.-T. (2019). Antibiotic Susceptibility Test with Surface-Enhanced Raman Scattering in a Microfluidic System. Anal. Chem..

[163] Liao C., Huang H., Chen Y., Huang N. The Microfluidic Microwell Device Integrating Surface Enhanced Raman Scattering for Bacteria Enrichment and in Situ Antibiotic Susceptibility Test. Proceedings of the 2020 IEEE 33rd International Conference on Micro Electro Mechanical Systems (MEMS).

[164] Bodelón G., Montes-García V., Pérez-Juste J., Pastoriza-Santos I. (2018). Surface-Enhanced Raman Scattering Spectroscopy for Label-Free Analysis of *P. aeruginosa Quorum* Sensing. Front. Cell. Infect. Microbiol..

[165] Lee W.-B., Chien C.-C., You H.-L., Kuo F.-C., Lee M.S., Lee G.-B. (2019). An integrated microfluidic system for antimicrobial susceptibility testing with antibiotic combination. Lab Chip.

[166] Ma L., Petersen M., Lu X. (2020). Identification and Antimicrobial Susceptibility Testing of *Campylobacter* Using a Microfluidic Lab-on-a-Chip Device. Appl. Environ. Microbiol..

[167] Tang Y., Zhen L., Liu J., Wu J. (2013). Rapid antibiotic susceptibility testing in a microfluidic pH sensor. Anal. Chem..

[168] Hu C., Kalsi S., Zeimpekis I., Sun K., Ashburn P., Turner C., Sutton J.M., Morgan H. (2017). Ultra-fast electronic detection of antimicrobial resistance genes using isothermal amplification and Thin Film Transistor sensors. Biosens. Bioelectron..

[169] Xu B., Du Y., Lin J., Qi M., Shu B., Wen X., Liang G., Chen B., Liu D. (2016). Simultaneous Identification and Antimicrobial Susceptibility Testing of Multiple Uropathogens on a Microfluidic Chip with Paper-Supported Cell Culture Arrays. Anal. Chem..

[170] He P.J.W., Katis I.N., Kumar A.J.U., Bryant C.A., Keevil C.W., Somani B.K., Mahobia N., Eason R.W., Sones C.L. (2020). Laser-patterned paper-based sensors for rapid point-of-care detection and antibiotic-resistance testing of bacterial infections. Biosens. Bioelectron..

[171] Bragazzi N.L., Amicizia D., Panatto D., Tramalloni D., Valle I., Gasparini R., Donev R. (2015). Chapter Six—Quartz-Crystal Microbalance (QCM) for Public Health: An Overview of Its Applications. Advances in Protein Chemistry and Structural Biology.

[172] Reyes P.I., Yang K., Zheng A., Li R., Li G., Lu Y., Tsang C.K., Zheng S.X.F. (2017). Magnesium Zinc Oxide Nanostructure-modified Quartz Crystal Microbalance for Dynamic Monitoring of Antibiotic Effects and Antimicrobial Resistance. Procedia Technol..

[173] Reyes P.I., Yang K., Zheng A., Li R., Li G., Lu Y., Tsang C.K., Zheng S.X.F. (2017). Dynamic monitoring of antimicrobial resistance using magnesium zinc oxide nanostructure-modified quartz crystal microbalance. Biosens. Bioelectron..

[174] Toosky M.N., Grunwald J.T., Pala D., Shen B., Zhao W., D’Agostini C., Coghe F., Angioni G., Motolese G., Abram T.J. (2020). A rapid, point-of-care antibiotic susceptibility test for urinary tract infections. J. Med. Microbiol..

[175] Abram T.J., Cherukury H., Ou C.Y., Vu T., Toledano M., Li Y., Grunwald J.T., Toosky M.N., Tifrea D.F., Slepenkin A. (2020). Rapid bacterial detection and antibiotic susceptibility testing in whole blood using one-step, high throughput blood digital PCR. Lab Chip.

[176] Wistrand-Yuen P., Malmberg C., Fatsis-Kavalopoulos N., Lübke M., Tängdén T., Kreuger J. (2020). A Multiplex Fluidic Chip for Rapid Phenotypic Antibiotic Susceptibility Testing. mBio.

[177] Choi J., Jung Y.-G., Kim J., Kim S., Jung Y., Na H., Kwon S. (2013). Rapid antibiotic susceptibility testing by tracking single cell growth in a microfluidic agarose channel system. Lab Chip.

[178] Baltekin Ö., Boucharin A., Tano E., Andersson D.I., Elf J. (2017). Antibiotic susceptibility testing in less than 30 min using direct single-cell imaging. Proc. Natl. Acad. Sci. USA.

[179] Li H., Torab P., Mach K.E., Surrette C., England M.R., Craft D.W., Thomas N.J., Liao J.C., Puleo C., Wong P.K. (2019). Adaptable microfluidic system for single-cell pathogen classification and antimicrobial susceptibility testing. Proc. Natl. Acad. Sci. USA.

[180] Adagio™ Adagio™ Antimicrobial Susceptibility Testing System. https://www.diagnostics-bio-rad.com/wp-content/uploads/2016/11/2015-Adagio-Brochure-EN.pdf.

[181] Strauss M., Zoabi K., Sagas D., Reznik-Gitlitz B., Colodner R. (2020). Evaluation of Bio-Rad® discs for antimicrobial susceptibility testing by disc diffusion and the ADAGIO™ system for the automatic reading and interpretation of results. Eur. J. Clin. Microbiol. Infect. Dis..

[182] Idelevich E.A., Becker K., Schmitz J., Knaack D., Peters G., Köck R. (2016). Evaluation of an Automated System for Reading and Interpreting Disk Diffusion Antimicrobial Susceptibility Testing of Fastidious Bacteria. PLoS ONE.

[183] Humphries R., Di Martino T. (2019). Effective implementation of the Accelerate Pheno™ system for positive blood cultures. J. Antimicrob. Chemother..

[184] Van Belkum A., Burnham C.-A.D., Rossen J.W.A., Mallard F., Rochas O., Dunne W.M. (2020). Innovative and rapid antimicrobial susceptibility testing systems. Nat. Rev. Microbiol..

[185] Barman P., Chopra S., Thukral T. (2018). Direct testing by VITEK(®) 2: A dependable method to reduce turnaround time in Gram-negative bloodstream infections. J. Lab. Phys..

[186] Lutgring J.D., Bittencourt C., McElvania TeKippe E., Cavuoti D., Hollaway R., Burd E.M. (2018). Evaluation of the Accelerate Pheno System: Results from Two Academic Medical Centers. J. Clin. Microbiol..

[187] Anton-Vazquez V., Adjepong S., Suarez C., Planche T. (2019). Evaluation of a new Rapid Antimicrobial Susceptibility system for Gram-negative and Gram-positive bloodstream infections: Speed and accuracy of Alfred 60AST. BMC Microbiol..

[188] Kost G.J. (2020). Geospatial Spread of Antimicrobial Resistance, Bacterial and Fungal Threats to COVID-19 Survival, and Point-of-Care Solutions. Arch. Pathol. Lab. Med..

[189] Burns J.L., Saiman L., Whittier S., Krzewinski J., Liu Z., Larone D., Marshall S.A., Jones R.N. (2001). Comparison of two commercial systems (Vitek and MicroScan-WalkAway) for antimicrobial susceptibility testing of *Pseudomonas aeruginosa* isolates from cystic fibrosis patients. Diagn. Microbiol. Infect. Dis..

[190] Yamakawa H., Kosai K., Kawamoto Y., Akamatsu N., Matsuda J., Kaku N., Uno N., Morinaga Y., Hasegawa H., Yanagihara K. (2018). Performance evaluation of BD Phoenix™, an automated microbiology system, for the screening of IMP-producing *Enterobacteriaceae*. J. Microbiol. Methods.

[191] Dickenson R.A., Chapin K.C. Comparative evaluation of the Sensititre ARIS 2X and the BD Phoenix automated identification and antimicrobial susceptibility test systems. Proceedings of the American Society for Microbiology Annual Meeting.

[192] MarketsandMarkets™ Antimicrobial Susceptibility Testing Market by Product (Manual, Automated Susceptibility Testing System), Type (Antibacterial, Antifungal), Application (Clinical Diagnostics), Method (ETEST, Disk Diffusion), End-User—Global Forecasts to 2025. https://www.marketsandmarkets.com/Market-Reports/antimicrobial-susceptibility-testing-market-206359984.html?gclid=Cj0KCQiA6t6ABhDMARIsAONIYyxnArdZQp3cfWqttKKKLOJw8d40ilYzt_KfHs0h_T9VYTzh652A6jMaAqgzEALw_wcB.

[193] MarketResearch.com. Antimicrobial Susceptibility Testing Market by Product (Manual Testing, Automated AST, Consumable), Method (Disk Diffusion, Dilution), Application (Clinical Diagnosis, Epidemiology), End User (Diagnostic Laboratories, Hospital)—Forecast to 2027. https://www.meticulousresearch.com/pressrelease/78/antimicrobial-susceptibility-testing-market-2027.

[194] El-Bouri K., Johnston S., Rees E., Thomas I., Bome-Mannathoko N., Jones C., Reid M., Ben-Ismaeil B., Davies A.P., Harris L.G. (2012). Comparison of bacterial identification by MALDI-TOF mass spectrometry and conventional diagnostic microbiology methods: Agreement, speed and cost implications. Br. J. Biomed. Sci..

[195] Moschou D., Tserepi A. (2017). The lab-on-PCB approach: Tackling the μTAS commercial upscaling bottleneck. Lab Chip.

[196] Tacconelli E., Sifakis F., Harbarth S., Schrijver R., van Mourik M., Voss A., Sharland M., Rajendran N.B., Rodríguez-Baño J. (2018). Surveillance for control of antimicrobial resistance. Lancet Infect Dis..

